# A STAT3 degrader demonstrates efficacy in venetoclax resistant acute myeloid leukemia

**DOI:** 10.1038/s41375-026-02883-9

**Published:** 2026-02-17

**Authors:** Samarpana Chakraborty, Claudia Morganti, Kimberly Zaldana, Bianca Rivera Pena, Hui Zhang, Divij Verma, Nadege Gitego, Feiyang Ma, Srinivas Aluri, Kith Pradhan, Shanisha Gordon-Mitchell, Ioannis Mantzaris, Mendel Goldfinger, Eric Feldman, Kira Gritsman, Yang Shi, Stefan Hubner, Yi Hua Qiu, Brandon D. Brown, Abdullah Khasawneh, Anna Skwarska, Eduardo Sabino de Camargo Magalhães, Amit Verma, Marina Konopleva, Yoko Tabe, Evripidis Gavathiotis, Simona Colla, Jared Gollob, Joyoti Dey, Steven M. Kornblau, Sergei B. Koralov, Keisuke Ito, Aditi Shastri

**Affiliations:** 1 Montefiore Einstein Comprehensive Cancer Centre (MECCC), Bronx, NY USA; 2 Department of Medicine (Oncology), Albert Einstein College of Medicine, Bronx, NY USA; 3 Department of Cell Biology, Albert Einstein College of Medicine, Bronx, NY USA; 4 Department of Pathology, NYU Langone Health, Bronx, NY USA; 5 Department of Biochemistry, Albert Einstein College of Medicine, Bronx, NY USA; 6 Department of Cell and Development Biology, Feinberg School of Medicine, Northwestern University, Chicago, IL USA; 7 Department of Pathology, Montefiore Medical Center, Bronx, NY USA; 8 Department of Leukemia, MD Anderson Cancer Center, Houston, TX USA; 9 Department of Clinical Laboratory Medicine, Juntendo University Graduate School of Medicine, Tokyo, Japan; 10 Department of Ageing Biology/ERIBA, University of Groningen, University Medical Center Groningen, Groningen, the Netherlands; 11 Kymera Therapeutics, Watertown, MA USA

**Keywords:** Acute myeloid leukaemia, Acute myeloid leukaemia

## Abstract

Acute myeloid leukemia (AML) is an aggressive myeloid malignancy with a poor prognosis. Venetoclax (Ven), a BCL2 inhibitor, has shown promising results but often leads to relapse due to mitochondrial dysregulation, particularly due to upregulation of the anti-apoptotic protein MCL1. Overexpression of the transcription factor STAT3 has been linked to poor survival in AML patients. Overexpression of STAT3 in a transgenic murine model induces a myeloid malignancy with a short latency period and inflammatory upregulation. The current study identifies STAT3 upregulation as a key mechanism of Ven resistance. A clinically relevant STAT3 degrader effectively reduces both total and phosphorylated STAT3, corrects mitochondrial structural and functional dysregulation, and induces apoptosis in Ven-resistant AML cell lines. KT-333 significantly decreases STAT3 and MCL1 protein levels and improves survival in Ven-resistant (Ven-Res) AML murine models. In summary, STAT3 hyperactivation is leukemogenic, is further potentiated in Ven-resistance and can be clinically targeted with a novel and specific STAT3 degrader.

**Figure:**
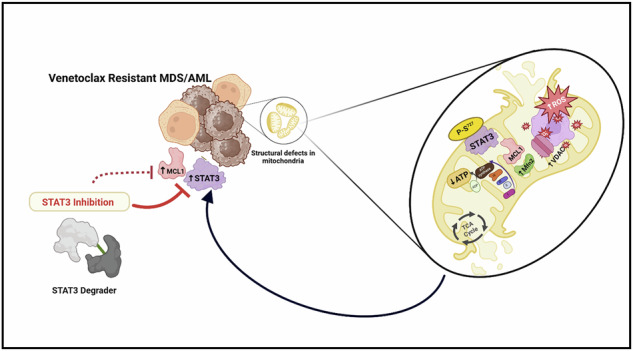
Pictorial representation depicting upregulation of STAT3 and MCL1 in venetoclax resistant myeloid malignancies such as MDS and AML causing mitochondrial structural abnormalities and dysfunction. By using specific STAT3 degrader, STAT3 inhibition, and thereby indirect downregulation of MCL1 can be a promising therapeutic intervention to target drug resistant clones in MDS and AML.

Pictorial representation depicting upregulation of STAT3 and MCL1 in venetoclax resistant myeloid malignancies such as MDS and AML causing mitochondrial structural abnormalities and dysfunction. By using specific STAT3 degrader, STAT3 inhibition, and thereby indirect downregulation of MCL1 can be a promising therapeutic intervention to target drug resistant clones in MDS and AML.

## Introduction

Acute Myeloid leukemia (AML) arises from malignant transformation of an immature hematopoietic precursor followed by clonal proliferation and accumulation of the transformed cells [1]. Ven is a selective inhibitor of the anti-apoptotic BCL2 protein and is now used extensively in the treatment of all frontline AML in combination with AML induction therapies such as Ven-FLAG-IDA (Combination of Fludarabine, Ara-C (cytarabine), G-CSF (granulocyte colony-stimulating factor) and Idarubicin), Ven 7 + 3 [2, 3]. While these therapies are highly effective for inducing remissions, the 5-year survival in newly diagnosed AML remains dismal at 30%, with disease relapse being inevitable due to the outgrowth of therapy-resistant hematopoietic stem and progenitor cells (HSPCs) [4].

Relapse or refractory disease after exposure to Ven is a particularly pervasive clinical problem and currently, there are no approved therapies that have demonstrated efficacy after the failure of Ven. Signal transducer and activator of transcription 3 (STAT3) belongs to the STAT family of transcription factors and is aberrantly activated in several malignancies [5, 6]. STAT3 phosphorylation at Y705 leads to its translocation to the nucleus where it is implicated in mediating oncogenic processes critical to cellular growth, proliferation and transformation [7]. Additionally, a pool of STAT3 (known as MitoSTAT3), is present in the mitochondria and undergoes phosphorylation at S727 residue which regulates the activity of the electron transport chain (ETC) and cellular respiration. However, the mitochondrial role of phosphorylated STAT3 (p-STAT3) is poorly understood in AML. While STAT3 has been an attractive target of research for therapeutic development for decades, the lack of specificity has led to off target effects and poor bioavailability of previously developed STAT3 inhibitors leading to their failure in clinical trials [8].

We have previously demonstrated that STAT3 is frequently de-methylated and overexpressed in myelodysplastic neoplasia (MDS) & AML HSPCs and is associated with an adverse prognosis [4]. We have also reported that STAT3 transcriptionally controls several important leukemic drivers that emerge during Ven-Res, most notably the anti-apoptotic protein myeloid cell leukemia-1 (MCL1). MCL1 overexpression is the main orchestrator of resistance to BCL2 inhibition in AML. MCL1 is a well-known direct transcriptional target of STAT3 and it is likely that changes to STAT3 affects downstream MCL1 activity [9].

We now present data that shows overexpression of STAT3 alone in a transgenic murine model induces a myeloid malignancy with a short latency period and inflammatory upregulation supporting STAT3’s role in inducing leukemogenesis. We also demonstrate that there is worse overall survival and reduced remission duration associated with total STAT3 and p-STAT3 upregulation in a large cohort of AML patients treated with Ven. Using multiple preclinical models, we show that total and p-STAT3 are upregulated in Ven-Res AML. Notably, the serine phosphorylated form of STAT3 is associated with mitochondrial dysfunction in Ven-Res AML. Importantly, a novel and clinically relevant STAT3 degrader effectively reverses the Ven-Res phenotype in AML cells, improving survival in a Ven-Res patient-derived xenograft (PDX) model and normalizing aberrant mitochondrial structure and function in leukemic HSPCs.

## Materials and methods

### Patient samples and reagents

MDS, AML and Venetoclax resistant AML patient samples were obtained from an IRB approved biobank at the Albert Einstein College of Medicine. Patient mutation profiles can be accessed from Supplementary Table S2. STAT3 degraders - KTX-201, KTX-105 and KT-333, along with structural controls were provided by Kymera Therapeutics. For in vitro studies, KTX-201 and KTX-105 were dissolved in DMSO to prepare 20 mM stocks. KT-333 was resuspended in PBS for in vivo murine cell-derived xenograft (CDX) and PDX studies.

### Cell lines

MOLM13, MOLM16 and SU-DHL1 cells were cultured in RPMI (Cytiva) with 10% fetal bovine serum (FBS, Gemini Bio-products) and 1% Penicillin-Streptomycin (Thermo Scientific). Venetoclax resistant cell line MOLM13 Ven-Res was generated as previously described [10]. MV411 cells were cultured in IMDM (Fisher Scientific) with 10% fetal bovine serum (FBS) and 1% Penicillin-Streptomycin (P/S). All cells were kept in a 5% CO2 37 °C incubator and regularly tested to be free of mycoplasma contamination.

### Mice

Our study examined male and female animals, and similar findings are reported for both sexes. NSG Mice were maintained under pathogen-free conditions in a barrier facility in microisolator cages based on a protocol approved by the Institutional Animal Care and Use Committee at Albert Einstein College of Medicine (AECOM).

### Generation of double transgenic STAT3C-vavCre mouse model

To determine the role of STAT3 in myeloid malignancies, a murine model was generated by crossing R26STAT3Cstopfl/fl mice with vavCre transgenic mice. R26STAT3Cstopfl/fl mice were generated and characterized as previously described [11].

### Real time PCR

RNA was isolated from frozen cell pellets using the RNeasy isolation kit (Qiagen) One microgram of total RNA was reverse-transcribed using the iScript cDNA Synthesis Kit (Bio-Rad). qPCR reactions were performed with the SYBR Green PCR Master Mix (Life Technologies) using validated gene- specific primers (IDT); (Supplementary Table S1). Transcript levels of genes of interest were normalized to a housekeeping gene (GAPDH) and fold change in expression was determined by –ΔΔCt, as previously published [9].

### Colony forming unit (CFU) assay

0.2–0.5 million Primary AML Peripheral Blood Mononuclear Cells (PBMNCs) and healthy controls were plated in Methocult media (Stem Cell Technologies H4435) in 35 × 10 mm dishes and incubated with and without 1 µM KTX-201. The cultures were incubated for 14–18 days. Erythroid and myeloid colonies were then counted, representative colony images were photographed (Nikon Eclipse Ts2), and the samples were processed for flow cytometry (BD LSR II), as previously performed [5].

### Electron microscopy

Cell lines and patient-derived PBMNCs were centrifuged at 1000 rpm for 10 min. This was followed by resuspension of the cells in 1 ml IMDM + 2% FBS. The resuspended cells were mixed with 1:1 ratio with fixative and submitted to EM core at Albert Einstein College of Medicine for imaging. The images were quantified manually and plots were prepared using GraphPad Prism software.

### BH3 profiling

MOLM13 Ven-Res cell lines were compared by BH3 profiling under basal condition by using the plate-based JC-1 BH3 profiling assay [12]. For dynamic BH3 profiling, cells were pre-treated with 100 nM KTX-105 for 24 h before profiling.

### Seahorse Mito Stress Test

The Seahorse XF96 Extracellular Flux analyzer (Seahorse Bioscience) was used to measure the oxygen consumption rate (OCR) of MOLM13 cells and MOLM13 Ven-Res cells according to the manufacturer’s instructions [13]. Briefly, AML cells were cultured with or without 100 nM KTX-201 for 24 h. Cells were counted, and 7.5 × 10^5^ cells were added to each well for the extracellular flux assays. Three technical replicates for each condition were plated.

### Statistical analyses

Data visualization was done using GraphPad Prism 10 software. For the comparison between two experimental groups, an unpaired *t* test was used. For more than two groups, ANOVA test was used with Tukey’s multiple comparisons test. In all graphs, error bars indicate mean ± SEM. A *P* value less than 0.05 was considered significant (*), with 0.01 (**), 0.001 (***), and 0.0001 (****) representing higher levels of significance. Non-normally distributed data was analyzed using Mann–Whitney’s test. Overall survival was measured and analyzed by Kaplan–Meier plotting using GraphPad Prism software. Immunoblot image quantitation was based on at least three independent biological replicates.

To perform this study, we have also generated cell derived as well as Ven-Res patient-derived xenograft models. Additionally, we have performed cell proliferation assays, western blots, caspase 3/7 assay, immunofluorescence, electron microscopy, Bulk-RNA seq, Single Cell RNA sequencing, ExCITE seq, subcellular fractionation and flow cytometry. Details of these methods are provided in supplementary.

## Results

### STAT3 overexpression drives myeloid bias

**Fig. 1:**
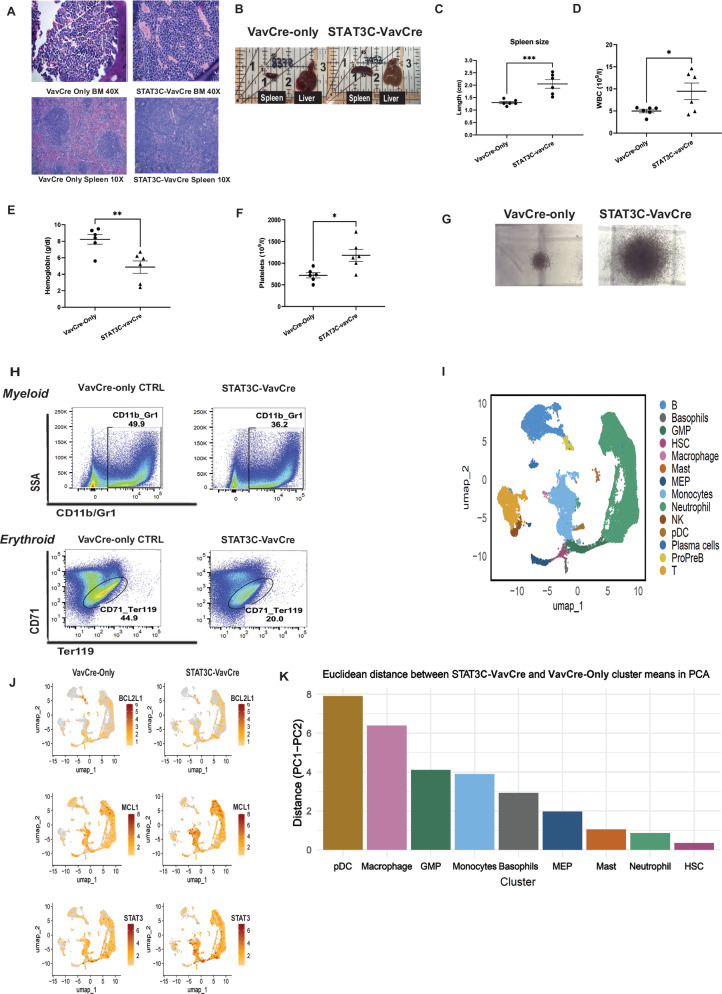
STAT3 overexpression drives myeloid bias. **A** Histopathologic image of the STAT3C-vavCre mice vs control shows complete destruction of splenic architecture with leukemic blasts. **B**, **C** Significant splenomegaly and hepatomegaly is observed in in STAT3C-vavCre mice (*n* = 6). **D**–**F** Complete Blood Count (CBC) analysis of STAT3C-vavCre mice showed increased WBC count, significant anemia and thrombocythemia, as compared to vavCre-only control mice (*n* = 6). **G** Representative image showing larger colonies in the STAT3C-vavCre mice compared to vavCre-only controls in murine myeloid colony assays. **H** FACS post CFU assay shows STAT3C-vavCre has a lower percentage of differentiated Myeloid and Erythroid cells compared to vavCre-only controls. **I**, **J** UMAP from ExCITE-Seq data shows upregulation of BCL2L1, MCL1 and STAT3 in Monocytes, HSC, GMP and neutrophils of STAT3C-vavCre mice as compared to vavCre-only control mice. **K** Principal component analysis (PCA) of ExCITE-seq data comparing STAT3C-VavCre and VavCre-only controls. Euclidean distances were calculated between STAT3C-VavCre and VavCre-only samples across hematopoietic clusters to evaluate population-level divergence in transcriptomic and surface protein profiles. Increased distances reflect greater dissimilarity between genotypes within a given cluster. Myeloid-specific clusters exhibited the greatest divergence, indicating a myeloid bias in STAT3C-VavCre samples.

To understand the role of STAT3 in initiation and progression of leukemia in the in vivo setting, we have developed a transgenic murine model with a hematopoietic-specific hyperactivation of the STAT3 pathway (STAT3C-vavCre model). The murine model was generated by crossing R26STAT3Cstopfl/fl mice with vavCre transgenic mice [11]. STAT3C-vavCre double transgenic mice (*n* = 17) were validated by GFP expression in HSPCs and differentiated hematopoietic cells [14]. The STAT3C-vavCre mice developed ruffled fur, a hunched phenotype and weight-loss at a mature age of 24–26 weeks of age [14]. Histopathologic analysis of the STAT3C-vavCre mice revealed complete destruction of splenic architecture with a spleen engorged with sheets of leukemic blasts and significant splenomegaly and hepatomegaly as compared to compared to vavCre-only controls (Fig. 1A–C). Complete Blood Count (CBC) analysis of STAT3C-vavCre mice showed significant anemia and progression to a proliferative phenotype with higher white blood cells (WBC) (Fig. 1D–F). CFU assay of the STAT3C-vavCre mice showed larger colonies along with lower percentage of differentiated erythroid and myeloid cells compared to controls (Fig. 1G, H). ExCITE-Seq data shows upregulation of STAT3 and markers of the apoptosis pathway [BCL2L1 (BCL-xL) and MCL1] in Monocytes, HSC, GMP and neutrophils of STAT3C-vavCre mice as compared to vavCre-only controls mice (Fig. 1I, J). Additionally, Euclidean distance between STAT3C-VavCre and vavCre-only control cluster in a PCA (Principal Component Analysis) space across the different hematopoietic clusters was calculated to assess population-level differences (Fig. 1K). Higher distances indicate greater transcriptional and surface protein divergence between STAT3C-VavCre and vavCre-only control samples for that particular cluster. Based on distance, we observe highest distance for myeloid-specific clusters on STAT3C hyperactivation, suggesting a myeloid bias. GSEA analysis showed an increase in expression of inflammatory pathways and decrease in erythrocytes in STAT3C-vavCre (STAT3C) mice as compared to vavCre-Only (WT) mice using ExCITE-Seq, that is typically observed in AML (Supplementary Fig. S1A–E).

### STAT3 is overexpressed in venetoclax resistant AML

**Fig. 2:**
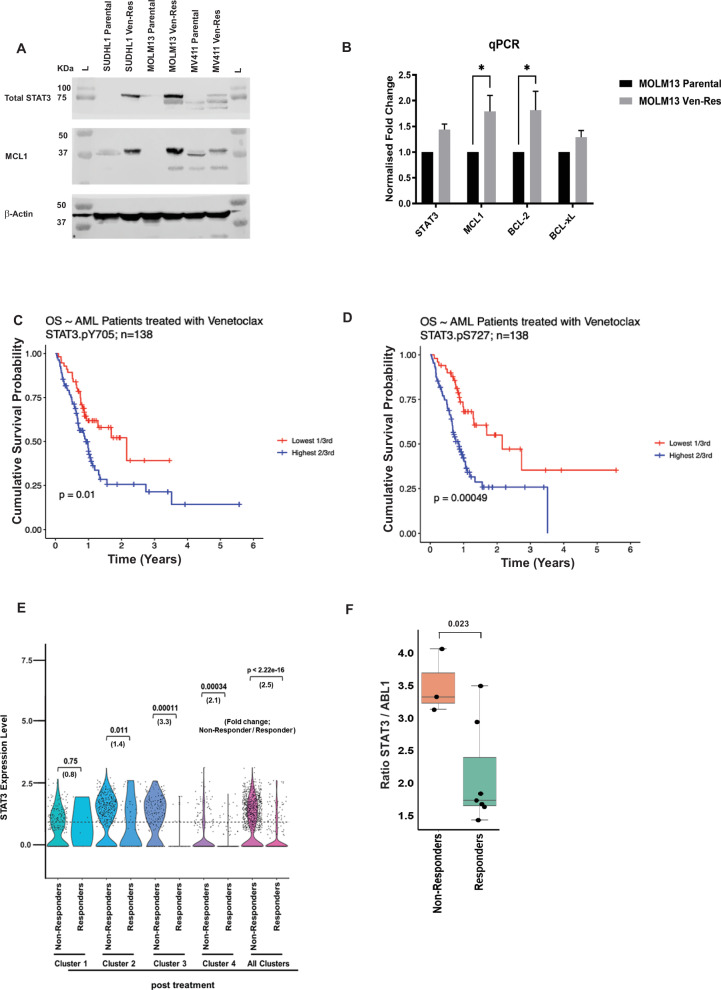
STAT3 is overexpressed in venetoclax resistant AML. **A** Increased expression of total STAT3 and MCL1 was observed in Ven-Res MOLM13, MV411 and SU-DHL1 cell lines as compared to their parental cell lines. **B** RT-PCR using RNA from parental and Ven-Res MOLM13 cell lines shows upregulation of total STAT3, MCL1, BCL2 and BCL-xL in MOLM13 Ven-Res cells. **C**, **D** Phospho-proteomic analysis on AML patients treated with Ven shows significant worse overall survival (OS) with higher expression of p-STAT3(Y705) and p-STAT3(S727), respectively. **E** Single cell RNA seq data on BM samples from post Ven/decitabine treated therapy-resistant relapsed/refractory AML patients shows higher expression of STAT3 as shown through fold change between non-responders vs responders. Cluster 1 corresponds to LSC-like cells, Cluster 2 to primitive-like cells, Cluster 3 to progenitor-like cells, and Cluster 4 to erythrocyte/monocyte-like AML cells. **F** Bulk RNA sequencing data from the same cohort at baseline show significantly higher STAT3 expression (*p* = 0.023) in non-responders as compared to responders.

We, and others have previously demonstrated that STAT3 is overexpressed in MDS/AML HSPCs [9, 15]. To understand the implication of Ven-Res in vitro, we developed Ven-Res AML cell lines – MOLM13, MV411 and lymphoma cell line SU-DHL1 by treating the parental cells with increasing dose of Ven over a period of 6-8 weeks. In the Ven-Res cell lines, we observed increased total STAT3 along with one of its downstream effector, MCL1, when compared to parental cell lines (Fig. 2A). qPCR validated our findings and additionally demonstrated increased expression of BCL2 and BCL-xL in MOLM13 Ven-Res cells (Fig. 2B). Furthermore, clinical data from AML patients that received treatment with Ven containing regimens has a significantly worse outcome if they had high expression of total STAT3 along with increased phosphorylation for  either p-STAT3(Y705) or p-STAT3(S727) for both overall survival (OS, *n* = 138 Y705 *p* = 0.01, S727 *p* = 0.0005) and remission duration (RemDur, *n* = 90, *p* < 0.05) (Fig. 2C, D; Supplementary Fig. S2 A, B). The data was further stratified based on treatment regimen, whether patients received Ara-C+ Ven, or Hypomethylating agents (HMA)+Ven (Supplementary Fig. S2 C-F). Kaplan–Meier (KM) survival curves were generated and statistical significance was determined using LogRank Test. Additionally, univariate and multivariate analysis using Cox proportional hazards model for each of the p-STAT3(Y705) and p-STAT3(S727) were performed. We demonstrate that among venetoclax treated patients, regardless of chemotherapy regimen (AraC or HMA), age, secondary AML status, or cytogenetic profile (including unfavorable, complex, or diploid karyotypes) - those with high expression of either p-STAT3(Y705) or p-STAT3(S727) exhibit significantly poorer clinical outcomes. Since both the p-STAT3 variables remained significant in the multivariate analysis, they can therefore be considered as independent predictors of disease outcomes (Supplementary Table S3). For statistical analysis, Kruskal–Wallis rank sum test (for continuous variables, such as age); Pearson’s Chi-squared test (for categorical variables, such as complex karyotype); or Fisher’s exact test (for categorical variables with small sample size) were performed. Single cell RNA seq using bone marrow (BM) sample from AML patients treated on a clinical trial with venetoclax and decitabine demonstrates elevated STAT3 expression in cellular clusters corresponding to primitive-like and progenitor-like AML cells that emerged in therapy-resistance/relapse post Ven and decitabine treatment (Fig. 2E, Supplementary Fig. S2G, H). Bulk RNA sequencing data from the same clinical trial demonstrates significantly higher STAT3 expression (*p* = 0.023) in non-responders as compared to responders, before Ven/decitabine treatment (Fig. 2F).

### Venetoclax resistance increases p-STAT3(S727) protein levels in vitro and leads to structural alterations in mitochondria

**Fig. 3:**
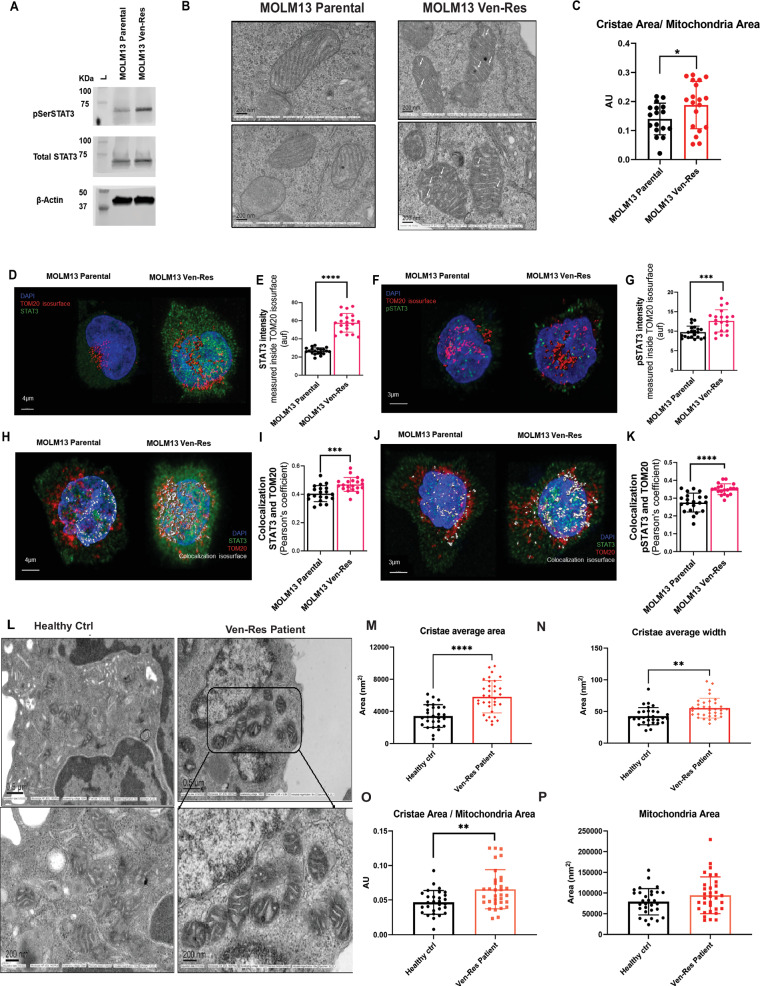
Overexpression of p-STAT3(S727) and mitochondrial structural alterations in Ven-Res AML. **A** Western blot on MOLM13 parental and Ven-Res cells shows increased expression of p-STAT3(S727) levels in Ven-Res. **B** EM imaging of MOLM13 Ven-Res cell lines show swollen node like structure in the mitochondria cristae of Ven-Res cells (shown in white arrows), as compared to MOLM13 parental cells. **C** Quantification of the EM image shows significant increase in cristae total area as compared to mitochondrial area in the MOLM13 Ven-Res cell line, as compared to MOLM13 parental cells. **D**–**K** Immunofluorescence image and their respective quantifications shows increased co-localization of total STAT3 and p-STAT3(S727) with TOMM20, in the mitochondria of MOLM13 Ven-Res cell line. TOMM20 is a protein that belongs to the import receptor complex of the mitochondrial outer membrane. Quantification of the IF signals shows a significant increase in the intensity of both total STAT3 and p-STAT3(S727) in the mitochondria with significant Pearson’s co- localization coefficient. **L** EM image of PBMNCs from Ven-Res patient shows huge nodule like cristae in mitochondria as compared to healthy subject (Healthy Ctrl). **M**–**P** Image analysis shows a significant increase in cristae area, width along with cristae total area as compared to mitochondrial area in the Ven-Res patient PBMNC sample as compared to healthy subject.

Previous report by Chen et al. have suggested possible structural and functional changes in mitochondria on Ven treatment [16]. The anti-apoptotic protein MCL1, known to localize in the mitochondria, is upregulated and plays a key role in the development of mitochondria mediated Ven-Res [9, 17, 18]. Additionally, literature also suggests presence of STAT3 in the mitochondria (MitoSTAT3) where it undergoes phosphorylation at Ser727 residue; p-STAT3(S727) [19]. MitoSTAT3 is known to be important for maintaining optimal mitochondrial respiration by modulating complex I and II of the ETC [19], and prevents ROS generated oxidative stress in the heart [20, 21]. It has been studied extensively in cancers such as chronic lymphoid leukemia, pancreatic and breast cancers [22–24], in addition to cardiovascular [21, 25] and neuronal research [26]. We hypothesized that in addition to MCL1, p-STAT3(S727) is also upregulated and causes mitochondrial dysregulation in Ven-Res AML. To confirm our hypothesis, we performed western blot on MOLM13 parental and Ven-Res cells and probed for p-STAT3(S727) levels. Ven-Res MOLM13 cells show an increased expression of p-STAT3(S727) on western blot as compared to MOLM13 parental cells (Fig. 3A). On comparing the ultrastructure of mitochondria using electron microscopy (EM), we observed swollen node-like structure of the cristae of MOLM13 Ven-Res cells, that were absent in the MOLM13 parental cells (Fig. 3B). Cristae are present in the inner membrane of the mitochondria where oxidative phosphorylation (OXPHOS)/ATP synthesis occurs and therefore, abnormalities in the cristae structure affect mitochondrial bioenergetics [27, 28]. Quantification of mitochondrial structure suggests an increased cristae total area as compared to the Ven-Res cell lines (Fig. 3C). We further checked if STAT3 localizes in the mitochondria using immunofluorescence, and observed a significant increase in the intensity of both total STAT3 and p-STAT3(S727) in the mitochondria (Fig. 3D–K). To validate our findings of mitochondrial structural abnormalities in primary patient samples, we collected fresh PBMNCs from Ven-Res patient samples and healthy subjects, fixed the cells and subjected them to EM imaging, followed by image analysis (Fig. 3L). The significant increase in cristae area, width along with cristae total area compared to mitochondrial area in the Ven-Res sample confirms our in vitro findings in primary patient samples (Fig. 3M–P).

### Novel STAT3 degraders effectively and selectively degrade STAT3, initiate apoptosis and reduce dependency on MCL1 for survival in Ven-Res AML

**Fig. 4:**
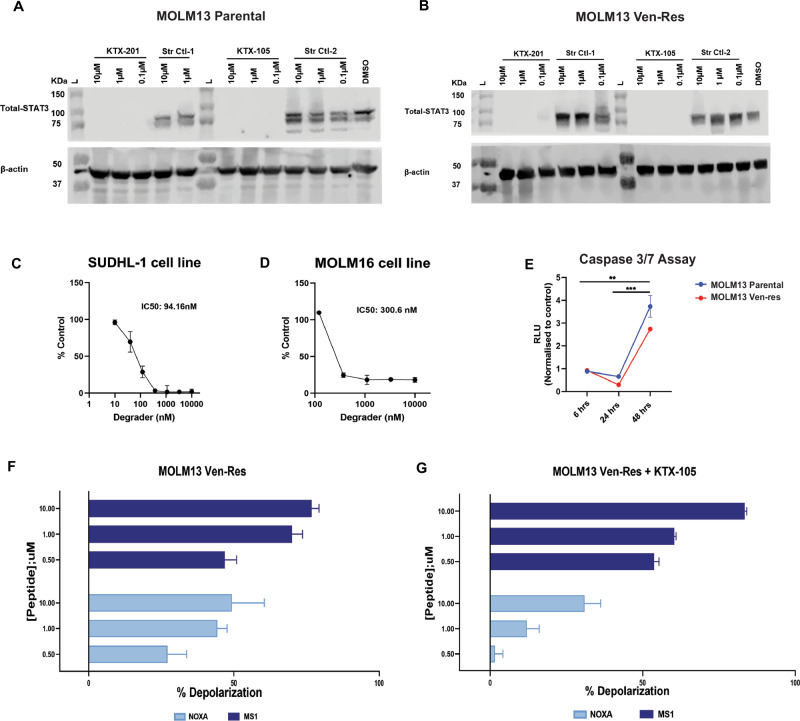
Selective degradation of STAT3 by degraders occur via initiating apoptosis and reduces dependency on MCL1 in Ven-Res AML. **A**, **B** Western blots showing selective degradation of STAT3 in both parental and Ven-Res MOLM13 cell line on treatment with STAT3 degraders (KTX-201, KTX-105) at 0.1, 1 and 10 μM doses for 24 h, with no effect on STAT3 levels when treated with structural controls (Str Ctl 1 and Str Ctl 2) or DMSO. **C**, **D** Dose dependent decrease in proliferation of SU-DHL1 and MOLM16 cell lines 72 h post treatment with KTX-201, as measured by CTG assay. **E** KTX-201 treatment led to significant induction of apoptosis in both MOLM13 parental and Ven-Res cell lines at 48 h, as measured by Caspase 3/7 assay. **F** BH3 profiling of MOLM13 Ven-Res cells shows depolarization when treated with MS1 and NOXA peptides even at 0.5 μM concentration, suggesting an increased dependency on MCL1 protein. **G** Treatment with KTX-105 led to reduction in NOXA induced mitochondrial depolarization in MOLM13 Ven-Res cell line.

As our data shows high STAT3 expression in Ven-Res AML, we hypothesized that STAT3 degradation can be a therapeutic strategy against Ven-Res AML. To this effect, we tested highly specific potent VHL (von Hippel-Lindau) based heterobifunctional tool degraders of STAT3 [29, 30] (KTX-201, KTX-105). It functions by forming a ternary complex involving STAT3, KTX, and the VHL E3 ligase, which facilitates rapid and site-specific ubiquitination of STAT3, leading to its subsequent proteasomal degradation [30]. The selective degradation of STAT3 in both parental and Ven-Res MOLM13 cell line was confirmed using western blot on treatment with two STAT3 degraders KTX-201 and KTX-105 at 0.1, 1 and 10 μM doses for 24 h, with no effect on STAT3 protein levels when treated with structural controls (Str Ctl-1, Str Ctl-2) or DMSO (Fig. 4A, B). Additionally, we confirmed these compounds selectively targeted STAT3 but not STAT5 (Supplementary Fig. S3A, B), suggesting target specificity [30]. Treatment with KTX-201 resulted in a dosedependent decrease in proliferation of cell lines that express high levels of STAT3 such as SU-DHL1 (lymphoma cell line) as well as MOLM16 (AML cell line) at nanomolar concentrations (Fig. 4C, D). KTX-201 treatment also led to significant induction of apoptosis in both MOLM13 parental and Ven-Res AML cell lines at 48 h (Fig. 4E). BH3 profiling of Ven-Res MOLM13 supports an increased dependency on MCL1 as observed by increase in mitochondrial depolarization on treatment with MS1 and NOXA peptides, which bind specifically to MCL1 (Fig. 4F). Interestingly, we observe that treatment with KTX-201 leads to more than 40% reduction in the mitochondrial depolarization of NOXA, thus reducing the dominion of MCL1 in mediating Ven-Res (Fig. 4G, Supplementary Fig. S3C, D).

### STAT3 degrader KTX-201 is taken up by primary AML and Ven-Res AML PBMNCs and leads to STAT3 downregulation and effective erythroid and myeloid differentiation

**Fig. 5:**
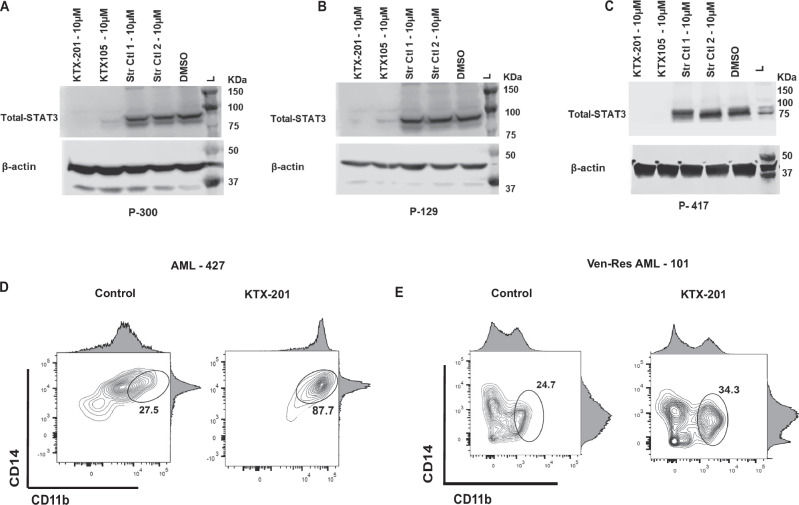
Primary AML and Ven-Res AML PBMNCS show effective degradation of STAT3 on treatment with KTX-201 and induce erythroid and myeloid differentiation. **A**, **B** Western blots showing effective degradation of total STAT3 in AML patient PBMNC and **C** Ven- res AML patient PBMNC samples on treatment with STAT3 degraders - KTX-201, KTX-105 for 24 h, with no change in STAT3 levels when treated with structural controls (Str Ctl1, Str Ctl 2) or DMSO. **D** FACS post CFU assay using AML patient PBMNC shows increased differentiation of myeloid markers CD11b and CD14, when treated with KTX-201. **E** FACS post CFU assay in Ven-Res AML patient PBMNC shows increased differentiation of myeloid markers CD11b and CD14, when treated with KTX-201.

In primary Ven-Res AML patient samples, we have noted effective degradation of STAT3 (>90%) in multiple patient PBMNC samples on treatment with KTX-201 and KTX-105 for 24 h (Fig. 5A–C), with no change in STAT3 levels when treated with structural controls (Str Ctl-1, Str Ctl-2) or DMSO. Stem and progenitor cells of MDS and AML show arrested and dysplastic differentiation [31, 32]. We treated primary AML and Ven-Res AML patient PBMNCs with KTX-201 and cultured them in methylcellulose assays supplemented with cytokines. Cells were harvested after 14 days and assessed for erythroid and myeloid differentiation by flow cytometry. We observed increased erythroid (~1.5 fold) and myeloid differentiation (~2.5 fold) on treatment with KTX-201 as measured by CD71, Glycophorin A that are markers of early erythroid differentiation and CD14, CD11b that are myeloid differentiation markers, especially in Ven-Res AML patients (Fig. 5D, E; Supplementary Fig. S4A, B). Interestingly, no differentiation was observed in healthy samples (Supplementary Fig. S4C), suggesting the specificity of the degrader to AML stem and progenitor cells with no effect on normal cell types.

### Treatment with STAT3 degrader can reduce total and MitoSTAT3 inside mitochondria and reverse mitochondrial dysfunction in Ven-Resistance

**Fig. 6:**
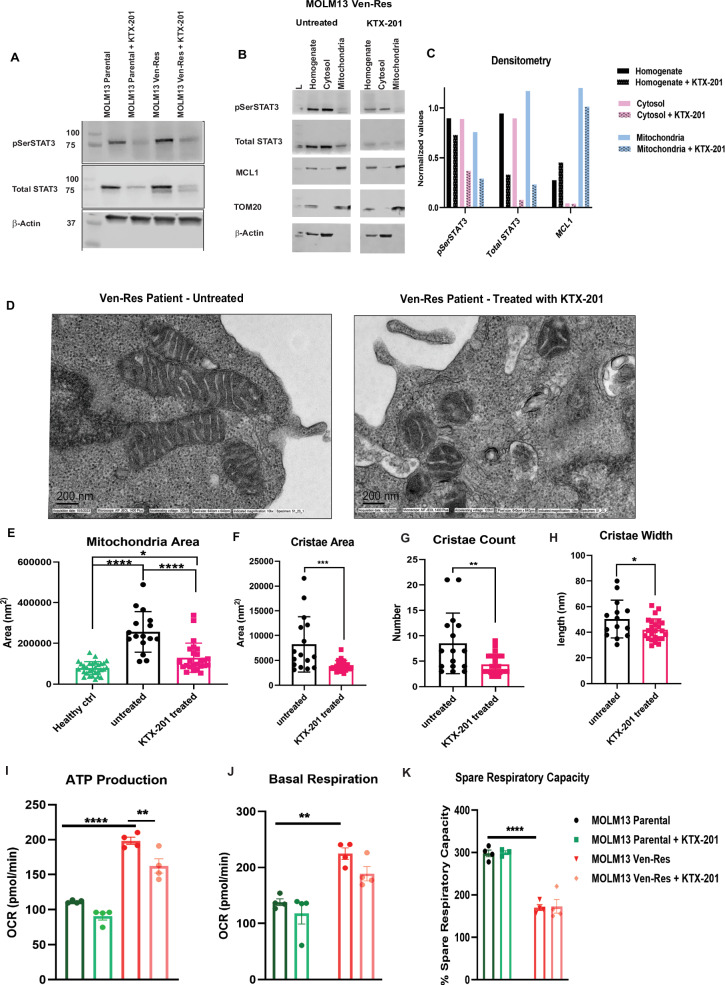
STAT3 degrader treatment can decrease MitoSTAT3 and mitigate mitochondrial dysfunction in venetoclax resistance. **A** Western blot showing significant reduction in the expression of p-STAT3(S727) in MOLM13 Ven-Res cells as compared to MOLM13 parental cells on treatment with 100 nM KTX-201 for 24 h. **B** Western blot on subcellular fractionated protein isolates from MOLM13 Ven-Res cell line, pre and post treatment with KTX-201, shows high expression of total STAT3 as well as p-STAT3(S727) in the mitochondria of the untreated cells, that undergo significant decrease on treatment with KTX-201. **C** Densitometry of the western blot show downregulation of STAT3, p-STAT3(S727) and MCL1, especially in the mitochondrial fraction post treatment with KTX-201. **D** EM imaging on PBMNCs from Ven-Res patient pre and post treatment with KTX-201 shows altered structure of mitochondria in the untreated sample suggesting fission/fusion defect, which reverses post treatment with KTX-201. **E** Image quantification of panel (**D**) shows significant decrease in mitochondrial area in Ven-Res sample post treatment with KTX-201, and is comparable to that of healthy control (Healthy Ctrl). **F**–**H** Treatment with KTX-201 also significantly reduced cristae area, cristae count and cristae width per mitochondria in Ven-Res PBMNC treated with KTX-201. **I**, **J** Mito stress test using MOLM13 parental and Ven-Res cells, shows an increase in ATP production and basal respiration in MOLM13 Ven-Res cells as compared to parental cells. Treatment with KTX-201 caused a significant decrease in ATP production and basal respiration in MOLM13 Ven-Res cells. **K** Mito stress test using MOLM13 parental and Ven-Res cells shows decrease in percentage (%) of spare respiratory capacity of MOLM13 Ven-Res cells as compared to parental cell line. Minor change in % spare respiratory capacity was observed post KTX-201 treatment.

After establishing that p-STAT3(S727) is upregulated in Ven-Res, we wanted to check if a STAT3 degrader can mitigate the damage caused by elevated MitoSTAT3. We observed that expression of p-STAT3(S727) undergoes significant reduction in MOLM13 Ven-Res cells as compared to MOLM13 parental cells on treatment with 100 nM KTX-201 (Fig. 6A). We hypothesize that on treatment with the STAT3 degrader, a global reduction in total STAT3 also greatly reduces the phosphorylated active forms of STAT3 in both mitochondria p-STAT3(S727) as well as cytoplasm p-STAT3(Y705). The resulting loss of STAT3 signaling are likely responsible for structural and functional changes in mitochondria. To understand if STAT3 degrader can reverse mitochondrial dysfunction in vitro, we performed subcellular fractionation using density gradient ultracentrifugation to isolate mitochondria in MOLM13 Ven-Res cell line, with and without treatment with KTX-201. Western blot of the protein isolates showed high expression of total STAT3 as well as p-STAT3(S727) in the mitochondria of the untreated cells, that undergo significant decrease on treatment with KTX-201 (Fig. 6B). Densitometry of the blot showed downregulation of STAT3, p-STAT3(S727) and MCL1 on treatment with KTX-201 (Fig. 6C). To ensure the observed mitochondrial changes are contributed by STAT3 specifically, MOLM13 Ven-Res cells were treated with 100 nM KTX-201 for 24 h, followed by media washout and collection of cells after an additional 120 h. Western blot analysis confirmed that the STAT3 degrader KTX-201 effectively reduced STAT3 protein levels at 24 h, whereas STAT3 expression returned to levels comparable to DMSO-treated control cells by 120 h (Supplementary Fig. S5A). Furthermore, electron microscopy (EM) and immunofluorescence (IF) analyses were performed to assess mitochondrial morphology. Using EM, we observed a marked decrease in mitochondrial cristae area, cristae width and cristae area/mitochondria area at 24-h post-treatment, which significantly recovered by 120 h. Morphologically, the mitochondria appeared more compact and structured at 24 h, but returned to a swollen, disrupted appearance by 120 h (Supplementary Fig. S5B–E). Additionally, using IF staining of mitochondrial protein TOMM20, we observed a marked reduction in total mitochondrial volume at 24 h along with a partial but significant increase by 120 h (Supplementary Fig. S5F). Furthermore, we isolated fresh PBMNCs from Ven-Res patients and fixed the cells pre and post treatment with KTX-201 and performed EM imaging. We observed long mitochondria in the untreated sample suggesting fission/fusion defects in the mitochondria, which post treatment with degrader leads to round and smaller fragments (Fig. 6D), suggesting possible priming of the cells towards apoptosis. Interestingly, image quantitation demonstrated that the decrease in mitochondrial area post treatment was comparable to that of healthy controls suggesting that STAT3 degradation can reverse the structural defects in mitochondria caused due to Ven-Res (Fig. 6E). Treatment with KTX-201 also reduced cristae count, cristae area and cristae width per mitochondria (Fig. 6F–H). To decipher the functional consequence of mitochondrial structural defect in Ven-Res, we performed functional analysis using Seahorse assay for MOLM13 parental and Ven-Res cells, pre and post treatment with KTX-201. MOLM13 Ven-Res cells showed a significant higher ATP production and basal respiration, suggesting they have higher metabolic activity as compared to parental cells [33]. This correlates with the concurrent increase in the cristae area of Ven-Res cells observed that signify an increase in mitochondrial respiration. However, a significant lower spare respiratory capacity of the resistant cells as compared to the parental cells suggests their declined ability to tolerate stress [34]. Treatment with KTX-201 caused a significant decrease in ATP production and basal respiration in MOLM13 Ven-Res cells (Fig. 6I–K).

### A significant increase in survival was observed on treatment with STAT3 degrader in PDX model of venetoclax resistance

**Fig. 7:**
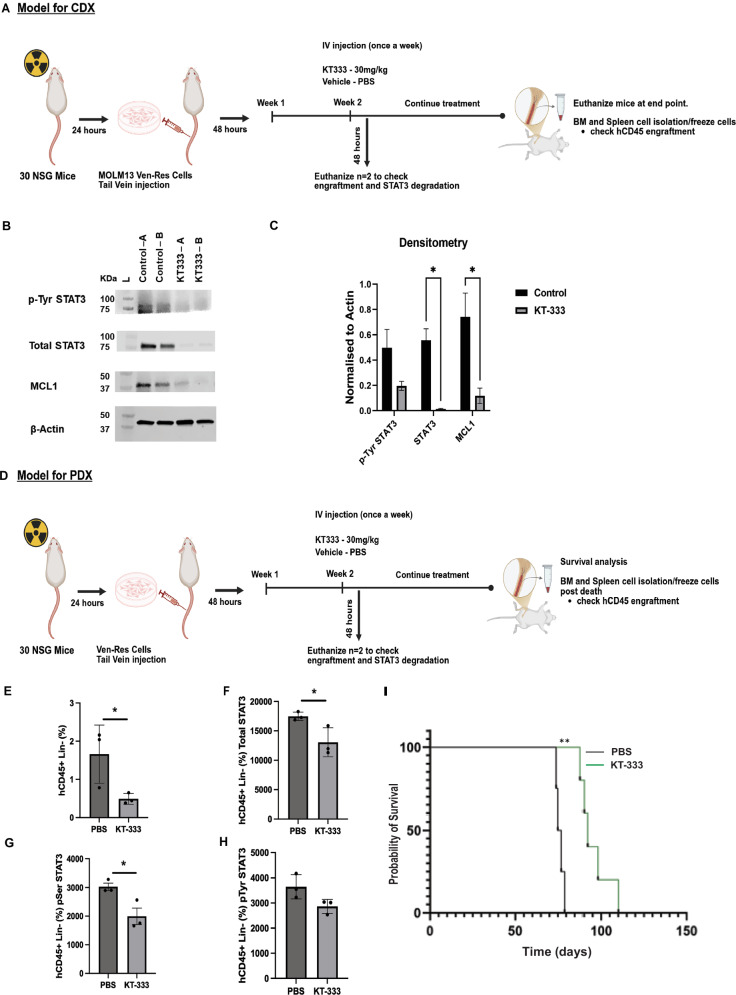
CDX and PDX model of venetoclax resistance show significant impact of STAT3 degrader in Ven-Res AML. **A** Schematic showing the development of cell-derived xenograft (CDX) model of systemic Ven-Res AML (*n* = 30). **B**, **C** Western blot and its densitometry showing significant reduction of p-STAT3(Y705), total STAT3 and MCL1 in spleen samples of control vs KT-333 treated mice, week 2 post-transplantation, 48 h post drug administration. **D** Schematic showing the development of patient-derived xenograft (PDX) model of systemic Ven-Res AML (*n* = 30). **E** Significant decrease in lineage negative HSPC population along with decrease in intracellular **F** total STAT3, **G** p-STAT3(S727) and **H** p-STAT3(Y705) is observed in the BM cells (*p* < 0.05) of PDX model collected on week 2, 48 h post treatment (*n* = 3). **I** Survival analysis of Ven-Res PDX model shows a significant improvement in survival of mice treated with KT-333 as compared to vehicle (Median survival 76 days in vehicle group vs 92 days in KT-333 treated cohort; *p* = 0.0027).

We evaluated the pharmacodynamic activity of KT-333, a clinical stage STAT3 degrader (currently in Phase 1 clinical trial: NCT05225584) in MOLM13 Ven-Res cells, a cell-derived xenograft (CDX) model of systemic Ven-Res AML (Fig. 7A). Spleens from these mice showed significant reduction of p-STAT3(Y705) (~60%), total STAT3 (>90%) as well as MCL1 (~70%), on treatment with two doses of KT-333, as assessed in week 2 post-transplant, 48 h post drug administration (Fig. 7B, C).

Next, to evaluate efficacy of KT-333 in Ven-Res AML, a murine patient-derived xenograft (PDX) model of Ven-Res systemic AML was developed (Fig. 7D) [35]. Briefly, NSG mice post irradiation were transplanted with patient-derived Ven-Res AML cells intravenously (IV). 48-hour post-transplantation, the mice were randomized into 2 groups to be treated with vehicle (PBS) or clinical stage STAT3 degrader KT-333 (30 mg/kg, IV) once a week. In BM aspirates collected on week 2, 48 h post treatment, we observed a significant decrease in lineage negative HSPC population along with decrease in intracellular total STAT3 and p-STAT3(S727) in the BM cells (*p* < 0.05) (Fig. 7E–H). Survival analysis shows a significant improvement in survival of mice treated with KT-333 as compared to vehicle (Fig. 7I; Median survival 76 days in vehicle group vs 92 days in KT-333 treated cohort; *p* = 0.0027).

## Discussion

Venetoclax resistance is a pervasive and challenging problem to overcome in AML therapy. As venetoclax becomes more widely used in the clinic, challenges related to resistance are likely to intensify. The median overall survival of AML patients after failure of Ven has been estimated to be 2.4–4 months [36]. While multiple biological mechanisms of Ven-Res have recently been elucidated [37], most of the approaches targeting these mechanisms have failed clinically or are currently too early in preclinical development [38].

There are several well characterized mutational drivers of AML and therapeutics targeting these mutations that are in clinical use, the best examples of which are the IDH1/2, FLT3 inhibitors [38–43]. However, these are not very efficacious as single agents and require combination with other therapies for enhanced efficacy [44, 45]. Lesser attention has been paid to clinically targeting downstream mediators of leukemogenesis and particularly transcription factors that are overexpressed in AML due to prior difficulties in developing effective drugs that can bind and downregulate or degrade the transcription factor of interest in leukemic HSPCs. However, targeting transcription factors represents an attractive and mutation agnostic path forward in AML therapeutics. Degrader technology is now coming of age across several oncologic and non-oncologic indications [46–49]. The enhanced specificity towards the protein of interest and selective targeting for degradation make the therapy especially attractive to spur further clinical development and test combinatorial approaches in both preclinical and clinical studies in AML.

This paper establishes several new paradigms on STAT3 hyperactivation in AML and shines light on a novel mechanism in AML and particularly therapy-resistant AML. STAT3 hyperactivation alone is sufficient to cause a myeloid malignancy at an older age with a significant expansion of the HSPC population and a de-differentiated phenotype in a transgenic murine model. STAT3 hyperactivation is compounded in the setting of therapy resistance in clinical patient datasets where prior exposure to Ven greatly upregulates STAT3 and its phosphorylated forms with a strong association to worsened overall survival. In addition, there is increased translocation of phosphorylated forms of STAT3 into Ven-Res mitochondria that correlate with structural and functional mitochondrial defects. A highly specific potent heterobifunctional degrader of STAT3 caused dose dependent and selective degradation of STAT3 as well as p-STAT3(Y705) and p-STAT3(S727) in both parental and Ven-Res hematological malignancy cell line. STAT3 degradation not only led to significant induction of apoptosis in cell lines, but also led to reduction in the MCL1 dependent mitochondrial depolarization as observed through BH3 profiling. Additionally, the structural defects in mitochondria were restored to levels comparable to healthy subjects upon treatment with the STAT3 degrader. In vivo patient-derived xenograft models of Ven-Res AML indicate that effective STAT3 degradation significantly enhances the survival of mice treated with KT-333. STAT3 degradation is a fresh and effective strategy with strong mechanistic rationale that can spur further clinical development of STAT3 degraders in Ven-Res AML.

One of the limitations of this study is that the bulk RNA-seq cohort included a small sample size that limits statistical power. In addition, heterogeneity in baseline disease characteristics, treatment history and molecular profiles may contribute to the observed variability in STAT3 expression. Additionally, we have not comprehensively explored the effects of all available leukemia therapeutics on mitochondrial structure. Our interest has been piqued by our findings of mitochondrial structural dysfunction after prior exposure to Ven. We have consistently identified these mitochondrial defects in Ven-Res cell lines as well as primary patient samples. These findings should be further validated in preclinical studies and ongoing clinical trials.

The advent of targeted therapies in AML has allowed more patients to proceed on a curative treatment path such as allogeneic stem cell transplantation. However, the significant concern for relapse brought on due to persistent abnormal hematopoietic stem cells still exists and rational stem cell targeting therapies are yet to make their way into the clinic. The approach to overcome venetoclax-induced therapy resistance by specifically targeting STAT3, an aberrant and upregulated transcription factor in leukemic stem cells, is novel and rapidly translatable to the clinic given that preliminary safety data already exists with the STAT3 degrader KT-333 [50, 51]. In addition, the strong upregulation of STAT3 in the setting of venetoclax resistance and the generation of a myeloid neoplasm in a transgenic murine model that hyperactivates STAT3 provide undeniable certainty that STAT3 is an important and effective target in the elusive pursuit of overcoming therapy resistance in high grade myeloid malignancies such as high risk MDS and AML.

## Supplementary information

Supplementary file

Supplementary Table S3

## Data Availability

The datasets are available from the corresponding author on reasonable request.
