# Supplementary material for: A STAT3 degrader demonstrates efficacy in venetoclax resistant acute myeloid leukemia
Source: Leukemia. 2026 Feb 17;40(4):717–29. doi: 10.1038/s41375-026-02883-9 (PMC13056550; doi:10.1038/s41375-026-02883-9)

Supplemental Information

A STAT3 Degrader Demonstrates Efficacy in Venetoclax Resistant Acute Myeloid Leukemia

Samarpana Chakraborty, Claudia Morganti*, Kimberly Zaldana*, Bianca Rivera Pena*, Hui Zhang, Divij Verma, Nadege Gitego, Feiyang Ma, Srinivas Aluri, Kith Pradhan, Shanisha Gordon, Ioannis Mantzaris, Mendel Goldfinger, Eric Feldman, Kira Gritsman, Yang Shi, Stefan Hubner, Yi Hua Qiu, Brandon D. Brown, Abdullah Khasawneh, Anna Skwarska, Eduardo Sabino de Camargo Magalhães, Amit Verma, Marina Konopleva, Yoko Tabe, Evripidis Gavathiotis, Simona Colla, Jared Gollob, Joyoti Dey, Steven M Kornblau, Sergei B. Koralov, Keisuke Ito, Aditi Shastri

The Supplemental information includes:

Materials and methods

Tables S1 and S2

Tables S3 – Excel file

Figures S1 to S5

Materials and Methods

Reverse Phase Protein Array measurement of total STAT3, p-STAT3(Y705) and p-STAT3(S727) Primary AML patient samples were enriched by ficoll separation and CD3/CD19 depletion, whole cell lysates prepared, then printed onto nitrocellulose slides, stained with highly validated antibodies, and analyzed as previously described (1). Pearson correlation between total, p-STAT3(Y705) and p- STAT3(S727) forms of STAT3 and the other 426 analyzed proteins was performed.

Generation of double transgenic STAT3C-vavCre mouse model

To determine the role of STAT3 in myeloid malignancies, a murine model was generated by crossing R26STAT3Cstopfl/fl mice with vavCre transgenic mice. R26STAT3Cstopfl/fl mice were generated and characterized as previously described (2). Briefly, Bruce4 C57BL/6 embryonic stem cells were transfected with a modified Rosa26 targeting vector that included a 59 floxed stop/Neo cassette and FLAG-tagged Stat3C cDNA, with an frt-flanked IRES-eGFP downstream (2). Excision of the stop cassette by Cre recombinase leads to expression of a flag-tagged STAT3C protein and concomitant expression of eGFP. R26STAT3Cstopfl/fl and Vav-iCre mice (The Jackson Laboratory) were crossed to obtain desired experimental genotype (R26STAT3Cstopfl/+ VavCre or R26STAT3Cfl/fl VavCre mice). All mice genotypes were determined by PCR and further confirmed by flow cytometry analysis on GFP expression in Hematopoietic stem cells (HSCs). PCR Primers used for mice genotyping are the following:

STAT3C forward 5'- GATGCAGTTTGGAAATAACGGTGAA-3';

STAT3C reverse 5'- GAGGTCAGATCCATGTCAAACGT-3';

Rosa26wt forward 5' TTCCCTCGTGATCTGCAACTC-3';

Rosa26wt reverse 5'-CTTTAAGCCTGCCCAGAAGACT-3';

Vav-iCre forward 5'- TCCTGGGCATTGCCTACAAC-3';

Vav-iCre reverse 5'-CTTCACTCTGATTCTGGCAATTTCG-3'.

Homozygous R26STAT3Cfl/fl VavCre mice were embryonically lethal, therefore heterozygous R26STAT3Cstopfl/+ VavCre mice were utilized for experimental purposes. For most experiments, control animals with a floxed stop cassette but lacking VavCre (R26STAT3Cstopfl/fl) and Vav-iCre control littermates were used. All mice were maintained on a clean C57BL/6 background and housed in specific pathogen-free conditions at the animal facility at Albert Einstein College of Medicine. Mice were handled by the animal facility staff and the designated laboratory technician. After sacrifice, sample processing was performed by team members blinded to treatment group allocation. No additional blinding was applied during data analysis. Animal care was within institutional animal care committee guidelines, and all experiments were performed in accordance with approved protocols for the Albert Einstein College of Medicine Institutional Animal Care and Usage Committee (IACUC).

<u>Characterization of double transgenic STAT3C-vavCre mice</u>

To determine the role of STAT3 in initiation or progression of myeloid malignancies, heterozygous R26STAT3Cstopfl/+ VavCre mice were utilized for experimental purposes. For most experiments, age- matched control animals with a floxed stop cassette without VavCre (R26STAT3Cstopfl/fl) and Vav- iCre control littermates were used. Bi-weekly CBC analysis and blood smears were performed on heterozygous R26STAT3Cstopfl/+ VavCre mice and control animals for detecting hematological abnormalities that may suggest an underlying malignancy. Mice displaying signs of a hematological malignancy on measuring CBC parameters such as low WBC counts, low platelets etc. in addition to decrease in mice weight, development of ruffled fur etc. were euthanized, followed by necropsy procedure. Bone

marrow, Spleen, and Liver tissues were harvested during necropsy and samples were further processed for histopathological analysis. Spleen organ length and weight were recorded.

ExCITE-seq for STAT3C-VavCre murine model

Spleen and bone marrow cells from the femur were collected from two wild-type and two STAT3C-VavCre mice. Each mouse's cells were individually hash-tagged using the 10X Chromium Next GEM Single Cell 5' Reagents Kits v2 Dual Index (CG000330 Rev C) as per the manufacturer's instructions. The cells were then counted using a NucleoCounter-300 automated cell counter with DAPI/AO dye. 2 million cells per sample were resuspended in CITE-seq staining buffer (2% BSA, .01% Tween in PBS) and incubated for 10 minutes with Fc receptor block (TruStain FcX, BioLegend and FcR blocking reagent, Miltenyi) to prevent antibody binding to Fc receptors. The cells were then incubated with hashing and ECCITE-seq surface panel antibodies for 30 minutes at 4°C. The antibodies used for cell hashing and our ECCITE-seq antibody-derived tags (ADT) panel were sourced as TotalSeq-C reagents (BioLegend). After staining, the cells were washed three times in PBS containing 2% BSA and .01% Tween, followed by centrifugation (300 ×g for 5 minutes at 4°C) and supernatant aspiration. After the final wash, the cells were resuspended in PBS and filtered through 40-µm cell strainers, combined into two lanes and loaded into the 10x Chromium Single Cell Immune Profiling workflow as per the manufacturer's instructions.

Post-emulsification, cDNA libraries underwent a serious of amplifications using the 10X Chromium Next GEM Single Cell 5' Reagents Kits v2 Dual Index (CG000330 Rev C) as per the manufacturer's instructions. Amplified cDNA's quality and quantity was assessed on an Agilent BioAnalyzer 210 using a High Sensitivity DNA Kit (Agilent Technologies) and the final libraries on an Agilent TapeStation 420 using High Sensitivity D1000 ScreenTape (Agilent Technologies). The individual libraries were diluted to 2nM and pooled for sequencing. The pools were sequenced with S1 100 Cycle Flow Cell v1.5 run kits (26bp Read1 and 91bp Read2)

on the NovaSeq 600 Sequencing System (Illumina), targeting 30,000 and 6,00 reads per cell for the GEX and ADT library, respectively. FASTQ files from the 10x libraries were processed using the count module of Cell Ranger pipeline, version 7.0.0. with Intron mode (10x Genomics) aligned to the mm10 ensemble.

<u>ExCITE-Seq Analysis</u>

Raw reads were processed using the Cellranger Pipeline (10x Genomics). The expression matrices for individual samples were merged using the Cellranger aggr function. The R package Seurat (v4.3.0) was used to cluster the cells in the merged matrix. Cells with less than 100 genes or more than 1e4 transcripts or 5% of mitochondrial expression were first filtered out as low-quality cells. The NormalizeData function was used to normalize the expression level for each cell with default parameters. The FindVariableFeatures function was used to select variable genes with default parameters. The ScaleData function was used to scale and center the counts in the dataset. Principal component analysis (PCA) was performed on the variable genes. The RunHarmony function from the Harmony package was applied to remove potential batch effect among individual samples. Uniform Manifold Approximation and Projection (UMAP) dimensional reduction was performed using the RunUMAP function. The clusters were obtained using the FindNeighbors and FindClusters functions. The cluster marker genes were found using the FindAllMarkers function. The cell types were annotated by overlapping the cluster markers with the published marker genes. The dot plot was plotted using the DotPlot function. The violin plots were plotted using the VlnPlot function. Differential expression analysis between two group of cells was conducted using the FindMarkers function. Genes with adjusted p value smaller than 0.05 were considered significantly differentially expressed. Enrichr was used for pathway enrichment analysis on the differentially expressed genes.

<u>Ven resistant Cell derived xenografts</u>

NOD/SCID IL2Rgamma KO NSG mice were initially purchased from Jackson Laboratory and then bred, housed, and handled in the animal facility of Albert Einstein College of Medicine. Animals were allocated to five mice per cage (two cages per group) based on sex, age, across groups. Randomization was performed by animal facility staff not involved in data analysis to minimize bias. NSG mice aged 8-10 weeks were sub-lethally irradiated (2.5 Gy) 24 hr prior to injection. 1 x 10^6 MOLM13 Ven-Res cells were administered via tail vein injection. 48-hour post-transplantation, the mice were divided into 2 groups to be treated with STAT3 degrader KT-333 (30mg/kg) or vehicle (PBS). Treatments were done once per week and BM aspirates were collected at week 2 post treatment. BM aspirates were used for flow cytometry based analysis using mice-CD45 FITC (Biolegend) 1:100, and human CD45 PerCPCy5.5 (Biolegend) to observe engraftment and western blot was performed to check for effective STAT3 degradation.

Ven resistant Patient Derived Xenografts

NOD/SCID IL2Rgamma KO NSG mice were bred, housed, and handled in the animal facility of Albert Einstein College of Medicine. NSG mice aged 8-10 weeks were sub-lethally irradiated (2.5 Gy) 24 hr prior to injection. Mononuclear cells from primary Ven-Res AML patients previously reported (3) were isolated by Ficoll separation. 1 × 10^5 MNCs were administered via tail vein injection. 3-4 weeks later, BM aspiration were performed and analyzed by flow cytometry for the human cell engraftment utilizing mice-CD45 FITC (Biolegend), and human CD45 PerCPCy5.5 (Biolegend). Mice were considered to be engrafted if they showed 0.1% or higher human derived CD45+ cells. The engrafted mice were randomized for treatment with 30mg/kg KT-333 or vehicle (PBS) once a week. Post 48 hours of treatment, BM aspirations and flow cytometry analysis were performed. Mice were considered severely ill and were euthanized upon reaching a moribund state. Their survival was recorded for the study and endpoint BM and spleen cells were collected and cryopreserved.

Drug Treatment of Mice

The mice were treated 30mg/kg KT-333 once a week intravenously. The control mice were treated with PBS once a week.

Bone Marrow Aspirates

For femoral bone marrow aspirations mice will be anesthetized. The animal's leg was first disinfected with 70% ethanol; a fine needle was then inserted into the femoral bone marrow cavity through the distal condyles. A small volume (up to 10 µL) of bone marrow was aspirated as this has been shown not to compromise the animal's functionality of the leg or their overall health. Sides (left/right) were alternately used for sequential aspirates. Animals received 5mg/kg Banamine pre-emptive per subcutaneous injection as an analgesic.

Cell Proliferation Assay

Cell lines and primary samples were incubated at concentrations of 100nM–10 µM of STAT3 degraders- KTX-201 and KTX-105 and structural controls. Cells were plated in four 96-well plates (1 x 10^4 cells/well) and treated with KTX-201 and KTX-105 in triplicate for 24 hours, 48 hours, 72 hours. The amount of ATP present was measured using CellTiter-Glo Luminescent Cell Viability Assay (CTG Assay, Promega). Cell Titer Glo reagent was added 1:1 before allowing the plate to gently rock 20 minutes at room temperature. Luminescence measured by a Fluostar Omega Microplate reader (BMG Labtech).

Immunoblotting

Cells were treated with STAT3 degrader (KTX-201, KTX-105, KT-333, Kymera Therapeutics, MA) or vehicle control for 24 hours. Cells were then centrifuged at 350xg for 5 minutes and pellets were collected. Protein lysates were prepared with 1% NP-40 lysis buffer (20 mmol/L Tris-HCl, pH 7.5; 150 mmol/L NaCl; 1 mmol/L EDTA; 150 mmol/l NaCl;1 % NP-40) containing protease inhibitors (Roche) and phosphatase inhibitors cocktail 2 and 3 (Sigma). The

cells were lysed for 30 to 45 minutes at 4°C followed by centrifugation at 14000 RPM for 40 minutes at 4°C. Protein quantification was performed using BCA assay. 60 μg of protein was resolved on SDS-PAGE (Biorad; 4–15% Mini-PROTEAN® TGX™ Precast Protein Gels) at constant voltage of 80-100V, followed by transfer to PVDF membrane (EMD-Millipore). Western blot analysis was performed with the following antibodies: STAT3 (Cell Signaling Technology), p-STAT3(Y705) (Cell Signaling Technology), pSTAT3(S727) (Cell Signaling Technology), MCL1 (BD biosciences) and β-actin (Santa-Cruz Biotechnology). Normalisation was done using BioRad Image Lab software.

Bulk RNA-seq

RNA sequencing libraries were prepared using the TruSeq Stranded Total RNA Ribo-Zero Gold Kit (Illumina) and sequenced on a NovaSeq 6000 system to generate 50-base paired-end reads. FASTQ files were quality-checked using FastQC, and only samples that passed QC were included in downstream analysis. Reads were aligned to the human reference genome (hg38) using STAR, and gene-level counts were obtained. Differential expression analysis was performed using the DESeq2 package, which included normalization, dispersion estimation, and statistical testing using the Wald test. Genes with a false discovery rate (FDR) < 5% were considered differentially expressed. Gene Set Enrichment Analysis (GSEA) was used for pathway-level evaluation, and gene sets were obtained from published signatures. Statistical significance in GSEA was defined as FDR < 0.25 based on 1,000 permutations.

Single cell RNA seq

Single-cell RNA-seq was performed on bone marrow samples using the 10x Genomics Chromium platform (3′ or 5′ kit), and libraries were sequenced with the DNBSEQ-G400 system. FASTQ reads were aligned to the GRCh38 human reference genome using Cell Ranger v7.0.1 to generate count matrices, which were analyzed using the Seurat package. Cells with low or

high gene counts (<200 or >3000 genes) and those with high mitochondrial content (>15%) were filtered out. Data were normalized, cell cycle effects regressed, and dimensionality reduced via PCA. Batch effects were corrected using Harmony. Cells were clustered using multiple resolution settings. Healthy controls were annotated via Azimuth. Uncharacterized AML clusters were identified using UMAP positioning and differentially expressed genes (DEGs) determined by Seurat's FindAllMarkers. Based on marker gene expression and UMAP, four AML clusters were defined: Cluster 1, LSC-like; Cluster 2, Primitive-like; Cluster 3, Progenitor-like; Cluster 4, Erythrocyte/monocyte-like.

Caspase 3/7 Assay

MOLM13 Parental and Ven-Res cells (5×10^3 cells/well) were seeded in a 96-well white plate and treated with 100nM KTX-201. Caspase 3/7 activation was measured after 6hr, 24hr and 48 hr by addition of the Caspase-Glo 3/7 reagent according to the manufacturer's protocol (Promega). Luminescence was detected by a microplate reader (TECAN). Caspase assays were performed in triplicate and the data normalized to vehicle-treated control wells.

Flow Cytometry

Human samples: Human CFU samples were counted after a 14-day incubation period. To prepare the CFU cultures in semisolid media, 3 mL of 2% fetal bovine serum in phosphate-buffered saline (2% FBS- PBS) was added, and the mixture was incubated at 37°C for 1 hour. After thorough mixing, the culture was transferred to a 15 mL falcon tube and centrifuged at 350 x g for 10 minutes at 4°C to pellet the cells. The pellet was then washed with 2% FBS-PBS and resuspended in 100 µL of Zombie-NIR (1:4000 diilution), followed by a 15-minute incubation in the dark at room temperature. After another wash with PBS, the pellet was resuspended in 50 µL of flow antibody cocktail and incubated in the dark at room temperature for 15 minutes. Following a final wash, the cells were resuspended in PBS, and flow cytometry

was conducted using a BD LSRII, with data analysis performed using FlowJo. Erythroid cells were analyzed by staining of unfractionated BMCs with conjugated antibodies against CD71, Glycophorin A, CD11b, CD14, CD233, CD34, CD45 and CD49d (Biolegend).

Murine samples: For mouse samples, cells were suspended in sterile fluorescence-activated cell sorting buffer PBS containing 0.5% bovine serum albumin and 2 mM EDTA and stained with indicated surface markers for 30 minutes at 4°C. All flow cytometry data were acquired on a BD LSRII and analysed using FlowJo. Chimerism in transplantations was assessed by staining PBMCs with conjugated antibodies against CD45.1-APC (Biolegend) and CD45.2-PerCP-Cy5.5 (Biolegend).

BH3 Profiling

MOLM-13 Ven-Res cell lines were compared by BH3 profiling under basal condition by using the plate-based JC-1 BH3 profiling assay (4). BIM, BID, PUMA, BMF-y, BAD, NOXA, HRK-y, FS1 and MS1 BH3 peptides at indicated concentrations; Puma2A peptide (final concentration of 25 μM); alamethicin (final concentration of 25 μM); CCCP (final concentration of 10 μM) were added to 15μL of JC1-MEB staining solution (20 ug/mL oligomycin, 20 μg/mL digitonin, 2 μM JC-1, 10μM 2-mercaptoethanol in MEB) in a black 384-well plate (Corning, Corning, NY, USA, CLS 3573) using Tecan D300E dispenser. The MEB buffer consisted of 150mM mannitol, 10mM HEPES-KOH pH 7.5, 50mM KCl, 0.02mM EGTA, 0.02mM EDTA, 0.1% BSA and 5mM Succinate. Single-cell suspensions were washed twice in PBS and resuspended in MEB at 4× their final density (2×10^4 cells/well). One volume of the 4× cell suspension was added to one volume of the JCI-MEB staining solution. This 2× cell/staining solution was incubated at RT in the dark for 10 min to allow cell permeabilization and dye equilibration. A total of 15 μL of the 2× cell/staining solution mix was then added to each treatment well of the plate. The fluorescence was measured immediately at 590 nm emission 545 nM excitation using the M1000 microplate reader (TECAN) at 30°C every 15 min for a total of 3 h. Percentage of

depolarization was calculated by normalization to the AUC of solvent-only control DMSO (0% depolarization) and the positive control CCCP (100% depolarization), as previously described(4). Bar graph represents the % of mitochondria depolarization of cells detected by JC-1 upon treatment of BH3-derived peptides, n=3. For dynamic BH3-profiling cells were pre-treated with 100nM KTX-105 for 24 hours before BH3 profiling.

Immunofluorescence

Cells were seeded on Lab-Tek™ II Chamber Slide (Thermo Fisher Scientific) coated with Retronectin (Clonetech). Samples were fixed by PAF 4% for 10 min at RT, and then permeabilized by Triton 0.25% for 10 min at RT. After blocking with BSA 2% for 30 min at RT, samples were incubated overnight at 4ºC with primary Antibodies (dilution 1:50). After washing, samples were incubated with donkey anti- mouse AlexaFluor546 (Invitrogen) and donkey anti-rabbit AlexaFluor488 (Invitrogen) for 1 hr at RT at 1:500 dilutions. After wash, samples were mounted using Vectashield, a mounting medium for fluorescence with DAPI (Vector). Mouse monoclonal anti-TOMM20 (Abnova), rabbit anti-STA3 (Cell Signaling Technology) or rabbit anti-p-STAT3(S727) (Cell Signaling Technology) were used as primary Antibodies. Z-stack were acquired on Leica Stellaris 8 Confocal equipped with 63X oil immersion lens. Stacks were deconvolved using Huygens Essential (SVI). Analysis and representative image renderings were obtained by Imaris 7 (Bitplane).

Subcellular fractionation

Cell fractionation was performed as previously described (5). Briefly, 10^9 cells were harvested in PBS and washed by centrifugation at 500g for 5 min with PBS. The cell pellet was suspended in homogenization buffer [225 mM mannitol, 75 mM sucrose, 30 mM Tris-HCl pH 7.4, 0.1 mM ethylene glycol-bis(β-aminoethylether)-N,N,N′,N′-tetraacetic acid (EGTA), and 1mM PMSF (phenylmethylsulfonyl fluoride)] and gently disrupted by Dounce homogenization. The

homogenate was centrifuged twice at 600g for 5 min at 4°C to remove nuclei and unbroken cells, and the resultant supernatant was centrifuged at 7,000g for 10 min at 4ºC to pellet crude mitochondria. The supernatant was centrifuged at 20,000g for 30 min at 4ºC. Further centrifugation of the supernatant at 100,000g (70- Ti rotor; Beckman) for 90 min at 4ºC resulted in the isolation of ER (pellet) and cytosolic fraction (supernatant). To purify mitochondria, the crude mitochondrial fraction was suspended in isolation buffer [250 mM mannitol, 5 mM HEPES pH 7.4, and 0.5 mM EGTA] and subjected to Percoll gradient centrifugation (30% v/v Percoll) in a 10 ml polycarbonate ultracentrifuge tube, at 95,000g (SW41 rotor; Beckman) for 30 min at 4ºC. This result in the formation of two rings. The upper one includes mitochondria associated membranes (MAMs) and the lower one isolated mitochondria. Mitochondria-containing ring was then washed by centrifugation at 6,300g for 10 min at 4ºC to remove the Percoll and finally suspended in isolation medium.

Supplementary Tables:

S1. RT-PCR Primer sequences

Target	Forward Seq	Reverse Seq
STAT3	5′- GAGAAGGACATCAGCGGTAAG-3’	5′- AGTGGAGACACCAGGATATTG- 3’
BCL-xL	5’- GGTCGCATTGTGGCCTTT – 3’	5’-TCCGACTCACCAATACCTGCAT-3’
MCL-1	5′-AAAGAGGCTGGGATGGGTTT-3’	5′- CAAAAGCCAGCAGCACATTC- 3’
BCL2	5′-GGCTGGGATGCCTTTGTG-3’	5′-GCCAGGAGAAATCAAACAGAGG-3’
GAPDH	5′-TGCACCACCAACTGCTTAGC-3′	5′- GGCATGGACTGTGGTCATGAG-3′

S2. Patient mutation profile

Patient Name	Mutation Profile
Ven-Res Patient	JAK2V617F mutation (33.3% VAF), FISH shows trisomy 8 and complex cytogenetics.
AML 427	JAK2 - VAF: 94%; IDH1 - VAF: 47%; NPM1 - VAF: 44%
Ven-Res 101	NPM1 - VAF: 20%
Sample 417	TET2 – VAF: 86% ; GATA2 – VAF: 44% ; CEBPA – 2 mutations – VAF: 29% (pathogenic); 32% (likely pathogenic)
Sample 300	NPM1 – VAF: 49% ; TET2 – VAF: 40%
Sample 129	FLT3 Internal Tandem Duplication with signal ratio of >1
Ven-Res PDX	CEBPA - frameshift mutation- VAF: 29, 31% ; GATA2 missense mutation – VAF: 44% and TET2 nonsense mutation; VAF: 86%

Supplementary Figure Legends:

Supplementary Figure S1. A) GSEA analysis of the ECCITE data shows significant upregulation of inflammatory pathways in STAT3C- vavCre mice. B) ECCITE seq data shows more number of myeloid and neutrophil cells as compared to other cell types in STAT3C-vavCre mice. B, C) STAT3C-vavCre mice also shows higher percentage of myeloid and neutrophil cells in STAT3C-vavCre mice as compared to WT mice, suggesting myeloid bias that is typically observed in AML. D, E) UMAP showing enriched clusters specific to myeloid and neutrophil cell types in STAT3C-vavCre mice as compared to WT mice.

Supplementary Figure S2. A, B) Phospho-proteomic analysis on AML patients treated with Ven shows significant reduction in remission duration (RemDur) in patients with high expression of p-STAT3(Y705), p-STAT3(S727) respectively. C-F) Stratification of the Ven treated AML patients based on chemotherapy regimen (Ara-C+Ven vs HMA+Ven) also shows significant reduction in OS and remission duration (RemDur) in patients with high expression of p-STAT3(Y705), p-STAT3(S727) respectively. G, H) Single Cell RNA seq data on BM samples from pre and post Ven/decitabine treated AML patients show upregulated STAT3 expression in refractory clusters.

Supplementary Figure S3. A) Western blot showing no effect on the expression of STAT5 in MOLM13 parental cells when treated with STAT3 degraders (D1: KTX-201, D2: KTX-105) and their structural controls (C1, C2) at 0.1, 1 and 10μM doses for 24 hours. B) Western blot showing no effect on the expression of STAT5 in MOLM13 Ven-Res cells when treated with STAT3 degraders KTX-201 and KTX-105 and their structural controls at 0.1, 1 and 10μM doses for 24 hours. C, D) BH3 profiling of MOLM13 Ven resistant cells pre and post treatment with KTX-105.

Supplementary Figure S4. A) FACS post CFU assay using AML patient PBMNC shows increased differentiation of early erythroid markers CD71 vs GlyA on treatment with KTX-201. B) FACS post CFU assay using Ven-Res AML patient PBMNC shows increased differentiation of early erythroid markers CD71 vs GlyA from 7.7% to 12.6% on treatment with KTX-201. C) No differentiation in erythroid markers CD71 and GlyA was observed in PBMNC sample from healthy subject on treatment with KTX-201.

Supplementary Figure S5. MOLM13 Ven-Res cells were treated with 100 nM KTX-201 for 24 hours, followed by media washout and collection after 120 hours, to assess STAT3-specific mitochondrial changes. A) Western blot confirmed STAT3 degradation at 24 hours with recovery to control levels by 120 hours. B-E) Electron microscopy (EM) showed reduced cristae area, cristae width and cristae area/mitochondria area at 24 hours, which recovered by 120 hours. F) Immunofluorescence (IF) of TOMM20 revealed a decrease in mitochondrial volume at 24 hours, followed by partial restoration by 120 hours.

References

1. de Camargo Magalhaes ES, Hubner SE, Brown BD, Qiu Y, Kornblau SM. Proteomics for optimizing therapy in acute myeloid leukemia: venetoclax plus hypomethylating agents versus conventional chemotherapy. Leukemia. 2024;38(5):1046-56.

2. Fogli LK, Sundrud MS, Goel S, Bajwa S, Jensen K, Derudder E, et al. T cell-derived IL-17 mediates epithelial changes in the airway and drives pulmonary neutrophilia. J Immunol. 2013;191(6):3100-11.

3. DiNardo CD, Verma D, Baran N, Bhagat TD, Skwarska A, Lodi A, et al. Glutaminase inhibition in combination with azacytidine in myelodysplastic syndromes: a phase 1b/2 clinical trial and correlative analyses. Nat Cancer. 2024;5(10):1515-33.

4. Ryan J, Letai A. BH3 profiling in whole cells by fluorimeter or FACS. Methods. 2013;61(2):156-64.

5. Wieckowski MR, Giorgi C, Lebiedzinska M, Duszynski J, Pinton P. Isolation of mitochondria-associated membranes and mitochondria from animal tissues and cells. Nat Protoc. 2009;4(11):1582-90.

Figure S1

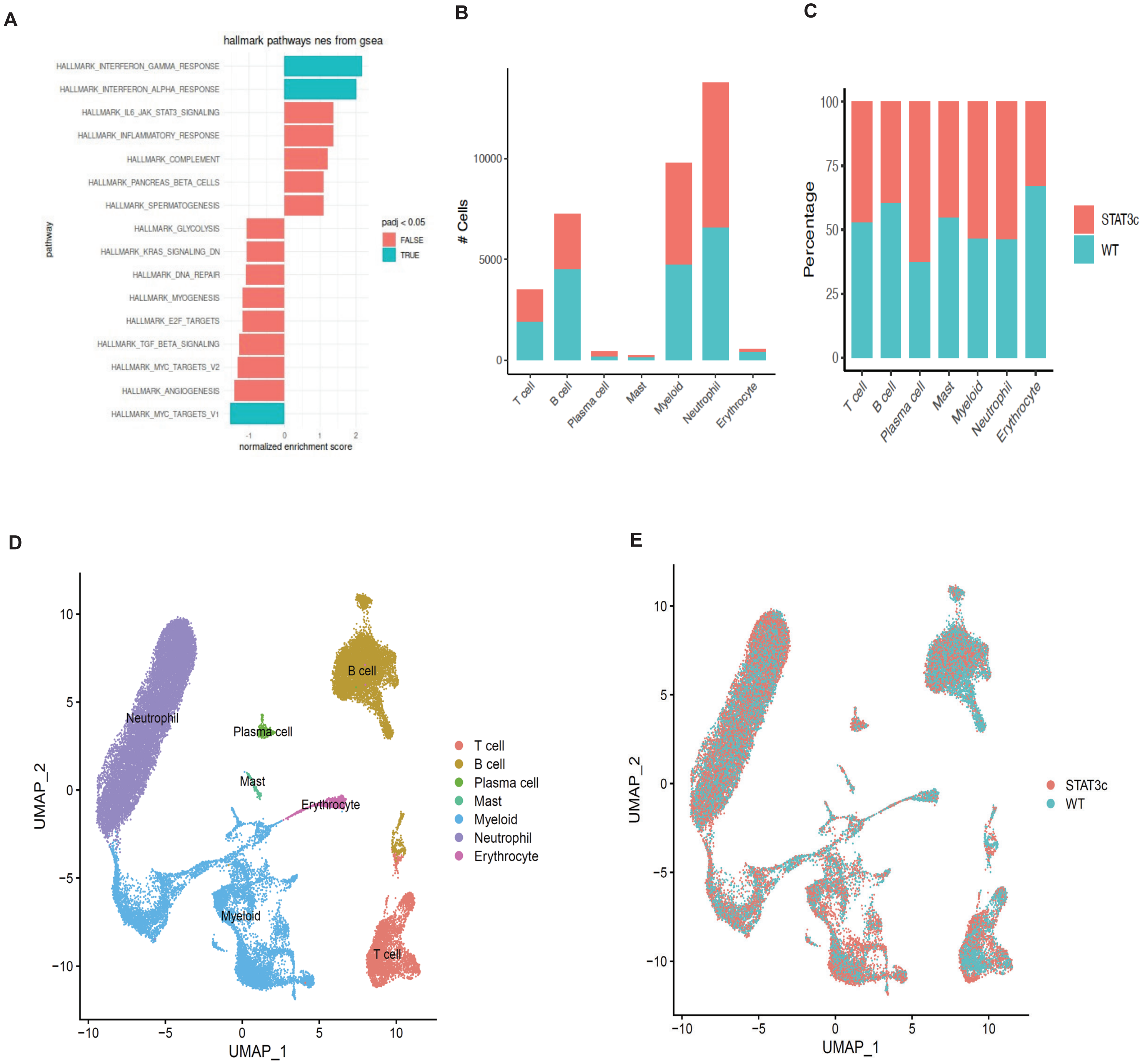
A
hallmark pathways nes from gsea
HALLMARK_INTERFERON_GAMMA_RESPONSE
HALLMARK_INTERFERON_ALPHA_RESPONSE
HALLMARK_IL6_JAK_STAT3_SIGNALING
HALLMARK_INFLAMMATORY_RESPONSE
HALLMARK_COMPLEMENT
HALLMARK_PANCREAS_BETA_CELLS
HALLMARK_SPERMATOGENESIS
HALLMARK_GLYCOLYSIS
HALLMARK_KRAS_SIGNALING_DN
HALLMARK_DNA_REPAIR
HALLMARK_MYOGENESIS
HALLMARK_E2F_TARGETS
HALLMARK_TGF_BETA_SIGNALING
HALLMARK_MYC_TARGETS_V2
HALLMARK_ANGIOGENESIS
HALLMARK_MYC_TARGETS_V1
pathway
padj < 0.05
FALSE
TRUE
normalized enrichment score
B
Cells
T cell
B cell
Plasma cell
Mast
Myeloid
Neutrophil
Erythrocyte
C
Percentage
STAT3c
WT
D
UMAP_1
UMAP_2
Neutrophil
Plasma cell
B cell
Mast
Erythrocyte
Myeloid
T cell
E
UMAP_1
UMAP_2
STAT3c
WT

Figure S2

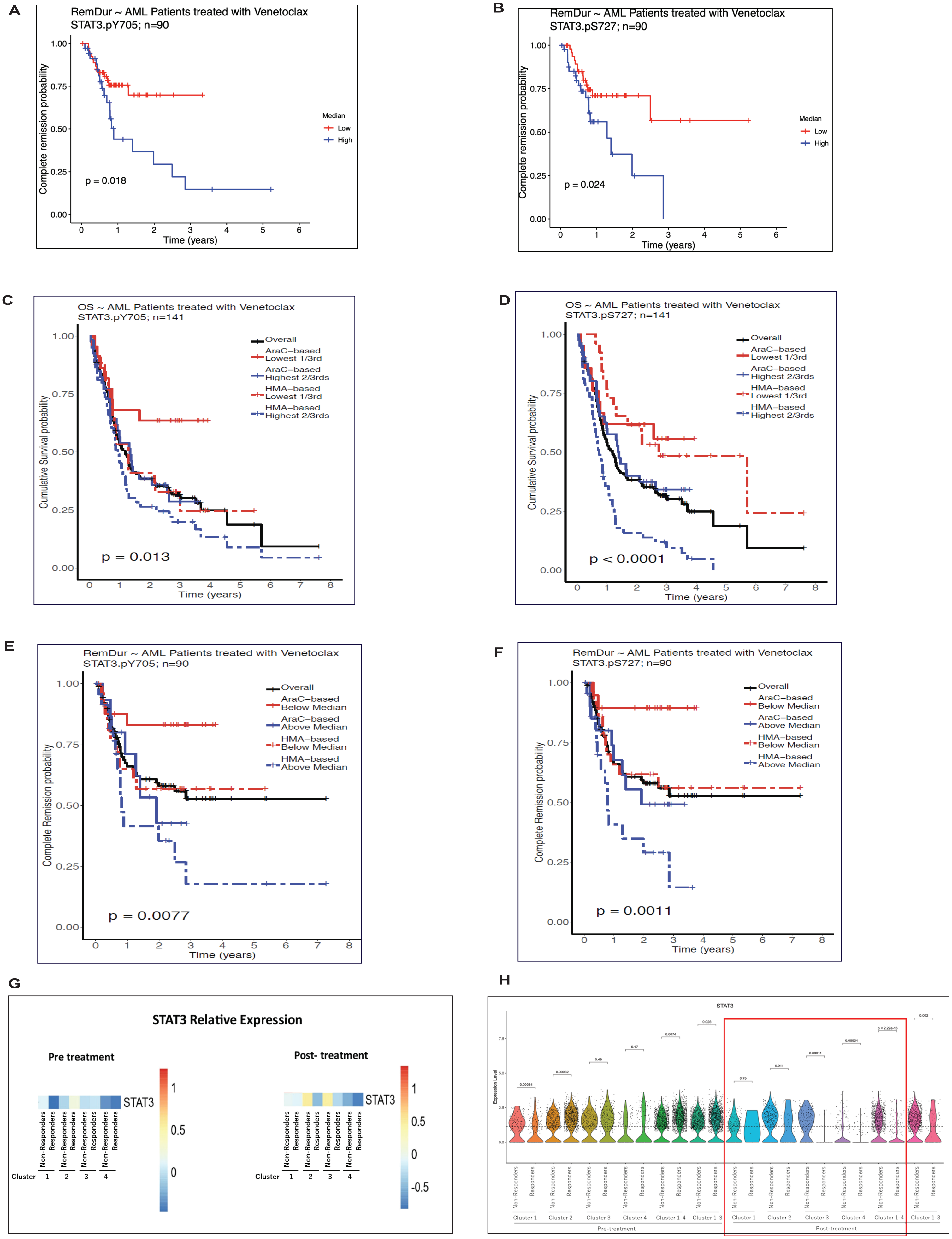
A
RemDur ~ AML Patients treated with Venetoclax
STAT3.pY705; n=90
Complete remission probability
Time (years)
Median
Low
High
p = 0.018
B
RemDur ~ AML Patients treated with Venetoclax
STAT3.pS727; n=90
Complete remission probability
Time (years)
Median
Low
High
p = 0.024
C
OS ~ AML Patients treated with Venetoclax
STAT3.pY705; n=141
Cumulative Survival probability
Time (years)
Overall
AraC–based Lowest 1/3rd
AraC–based Highest 2/3rds
HMA–based Lowest 1/3rd
HMA–based Highest 2/3rds
p = 0.013
D
OS ~ AML Patients treated with Venetoclax
STAT3.pS727; n=141
Cumulative Survival probability
Time (years)
Overall
AraC–based Lowest 1/3rd
AraC–based Highest 2/3rds
HMA–based Lowest 1/3rd
HMA–based Highest 2/3rds
p < 0.0001
E
RemDur ~ AML Patients treated with Venetoclax
STAT3.pY705; n=90
Complete Remission probability
Time (years)
Overall
AraC–based Below Median
AraC–based Above Median
HMA–based Below Median
HMA–based Above Median
p = 0.0077
F
RemDur ~ AML Patients treated with Venetoclax
STAT3.pS727; n=90
Complete Remission probability
Time (years)
Overall
AraC–based Below Median
AraC–based Above Median
HMA–based Below Median
HMA–based Above Median
p = 0.0011
G
STAT3 Relative Expression
Pre treatment
Post- treatment
STAT3
Non-Responders
Responders
Cluster 1 2 3 4
H
STAT3
Expression Level
Non-Responders
Responders
Cluster 1
Cluster 2
Cluster 3
Cluster 4
Cluster 1-4
Cluster 1-3
Pre-treatment
Post-treatment

Figure S3

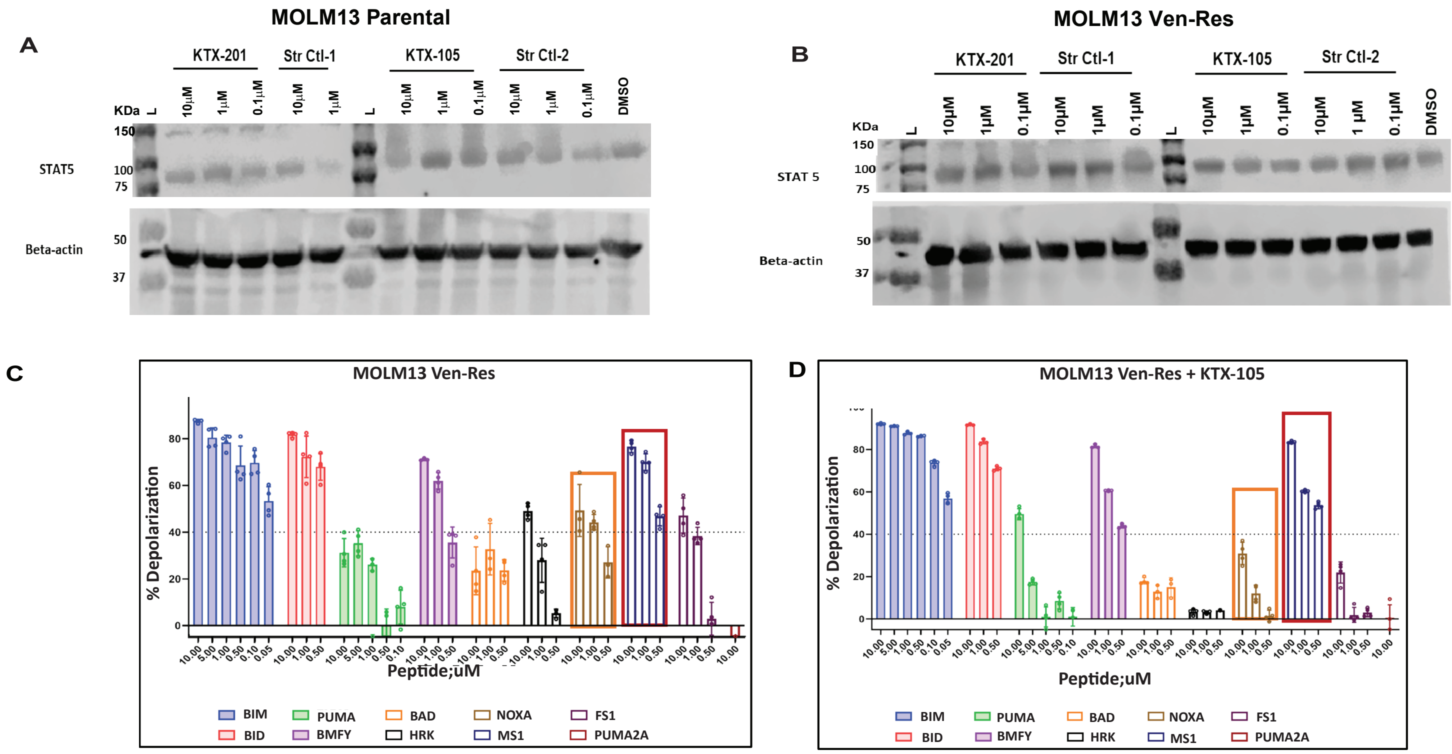

Figure S4

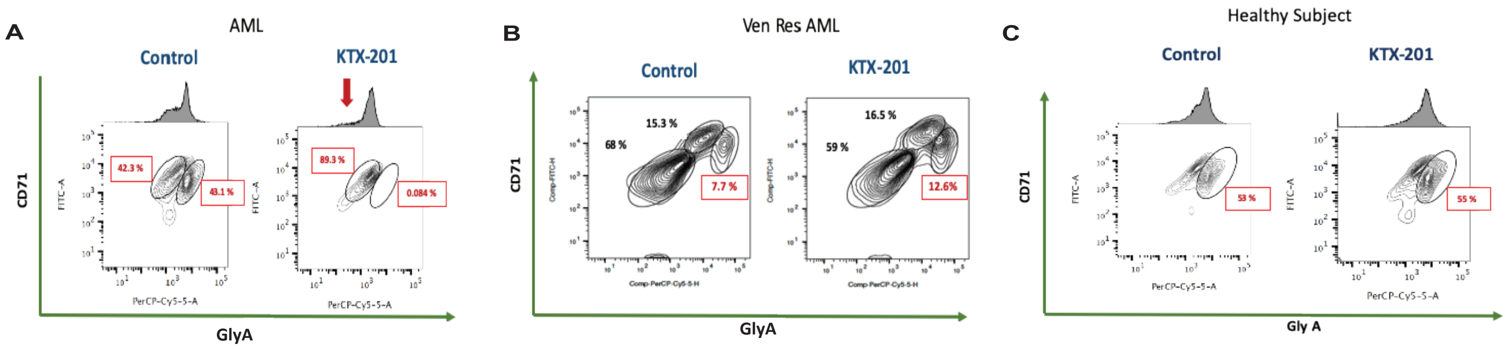

Figure S5

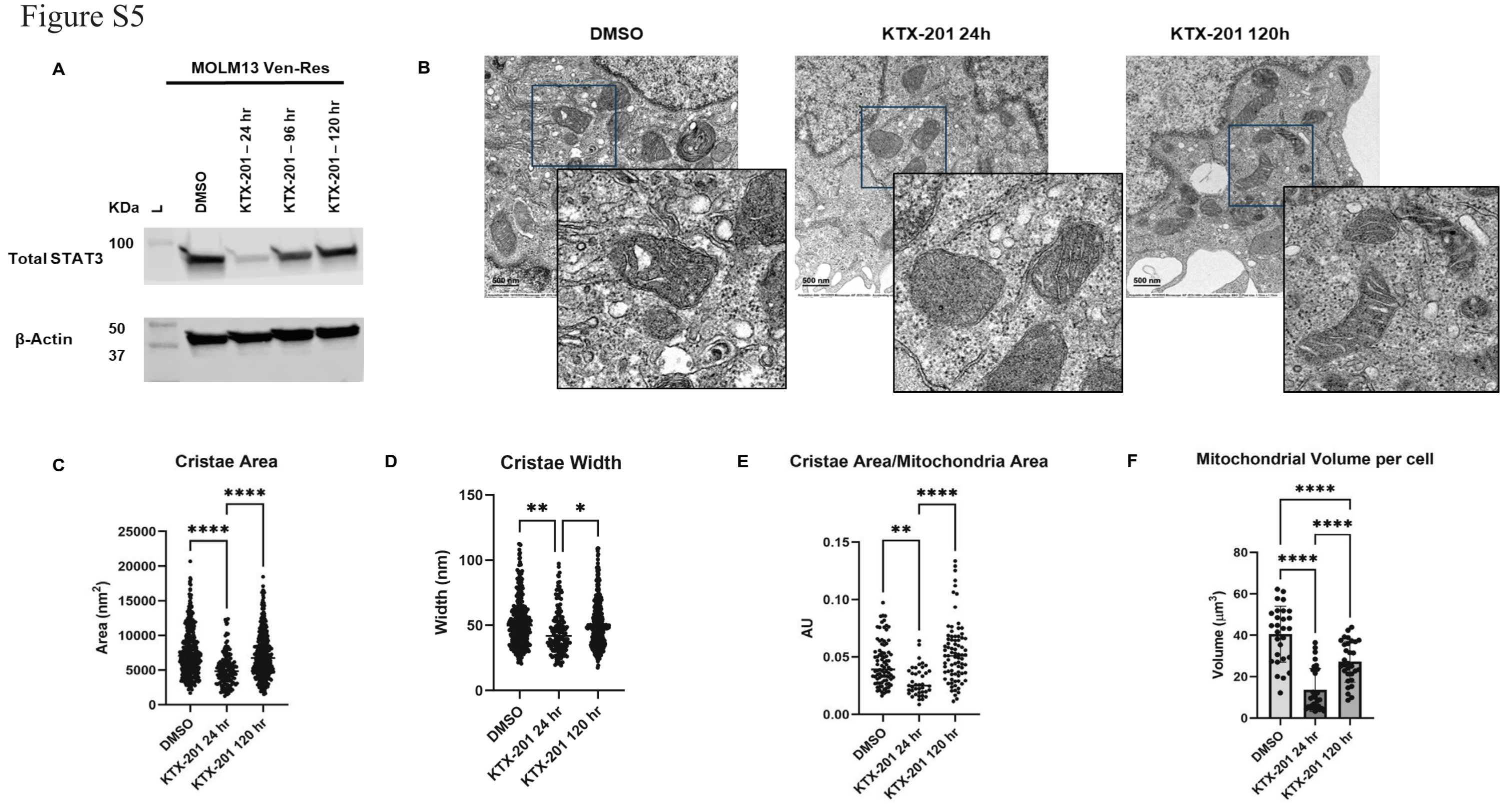

Supplement: Supplementary file 1 — Supplementary file [file 41375_2026_2883_MOESM1_ESM.pdf]
