# Supplementary material for: A STAT3 degrader demonstrates efficacy in venetoclax resistant acute myeloid leukemia
Source: Leukemia. 2026 Feb 17;40(4):717–29. doi: 10.1038/s41375-026-02883-9 (PMC13056550; doi:10.1038/s41375-026-02883-9)
Supplement: Supplementary file 2 — Supplementary Table S3 [file 41375_2026_2883_MOESM2_ESM.pdf]

Supplementary Table S3. Significant Univariate and Multivariate Cox Proportional Hazards models of Overall Survival in Ven treated patients

Variable	Univariate OS Ven treated (N=141)			Multivariate OS STAT3.pY705 Ven treated (N=141)			Multivariate OS STAT3.pS727 Ven treated (N=141)		
	HR	95% CI	p-value	HR	95% CI	p-value	HR	95% CI	p-value
STAT3.pY705 levels									
AraC-based Lowest 1/3rd	1.00	—		1.00	—				
AraC-based Highest 2/3rds	2.40	1.09, 5.28	**0.030**	3.16	1.39, 7.16	**0.006**			
HMA-based Lowest 1/3rd	2.34	1.01, 5.38	**0.046**	2.24	0.94, 5.36	0.069			
HMA-based Highest 2/3rds	3.28	1.54, 6.98	**0.002**	2.46	1.15, 5.29	**0.021**			
STAT3.pS727 levels									
AraC-based Lowest 1/3rd	1.00	—					1.00	—	
AraC-based Highest 2/3rds	1.69	0.79, 3.61	0.18				2.33	1.05, 5.20	**0.038**
HMA-based Lowest 1/3rd	0.96	0.41, 2.26	0.93				0.78	0.31, 1.99	0.60
HMA-based Highest 2/3rds	3.69	1.80, 7.55	**<0.001**				3.23	1.51, 6.90	**0.002**
Age (years)	1.02	1.00, 1.04	**0.010**						
Secondary AML	2.63	1.73, 4.01	**<0.001**	2.89	1.81, 4.62	**<0.001**	2.46	1.54, 3.93	**<0.001**
Unfavorable Cytogenetics	3.05	2.00, 4.66	**<0.001**						
Complex Karyotype	3.49	2.28, 5.36	**<0.001**	2.42	1.53, 3.85	**<0.001**	2.67	1.64, 4.36	**<0.001**
Diploid Karyotype	0.49	0.33, 0.75	**<0.001**	0.54	0.34, 0.85	**0.007**	0.61	0.39, 0.94	**0.026**
-5/5q-	2.52	1.57, 4.02	**<0.001**						
-7/7q-	2.31	1.40, 3.83	**0.001**	1.83	1.08, 3.12	**0.025**	2.00	1.16, 3.44	**0.013**
BCOR Mut	0.30	0.11, 0.84	**0.022**						
IDH2 Mut	0.25	0.11, 0.59	**0.001**						
NPM1 Mut	0.29	0.13, 0.64	**0.002**						
SRSF2 Mut	0.30	0.13, 0.66	**0.003**						
STAG2 Mut	0.12	0.02, 0.86	**0.035**						
TP53 Mut	3.38	2.08, 5.49	**<0.001**						

Abbreviations: CI = Confidence Interval, HR = Hazard Ratio
